# The changing global distribution and prevalence of canine transmissible venereal tumour

**DOI:** 10.1186/s12917-014-0168-9

**Published:** 2014-09-03

**Authors:** Andrea Strakova, Elizabeth P Murchison

**Affiliations:** 1 Department of Veterinary Medicine, University of Cambridge, Madingley Road, Cambridge CB3 0ES, UK

**Keywords:** Canine transmissible venereal tumour, Transmissible cancer, Epidemiology, Oncology

## Abstract

**Background:**

The canine transmissible venereal tumour (CTVT) is a contagious cancer that is naturally transmitted between dogs by the allogeneic transfer of living cancer cells during coitus. CTVT first arose several thousand years ago and has been reported in dog populations worldwide; however, its precise distribution patterns and prevalence remain unclear.

**Results:**

We analysed historical literature and obtained CTVT prevalence information from 645 veterinarians and animal health workers in 109 countries in order to estimate CTVT’s former and current global distribution and prevalence. This analysis confirmed that CTVT is endemic in at least 90 countries worldwide across all inhabited continents. CTVT is estimated to be present at a prevalence of one percent or more in dogs in at least 13 countries in South and Central America as well as in at least 11 countries in Africa and 8 countries in Asia. In the United States and Australia, CTVT was reported to be endemic only in remote indigenous communities. Comparison of current and historical reports of CTVT indicated that its prevalence has declined in Northern Europe, possibly due to changes in dog control laws during the nineteenth and twentieth centuries. Analysis of factors influencing CTVT prevalence showed that presence of free-roaming dogs was associated with increased CTVT prevalence, while dog spaying and neutering were associated with reduced CTVT prevalence. Our analysis indicated no gender bias for CTVT and we found no evidence that animals with CTVT frequently harbour concurrent infectious diseases. Vincristine was widely reported to be the most effective therapy for CTVT.

**Conclusions:**

Our results provide a survey of the current global distribution of CTVT, confirming that CTVT is endemic in at least 90 countries worldwide. Additionally, our analysis highlights factors that continue to modify CTVT’s prevalence around the world and implicates free-roaming dogs as a reservoir for the disease. Our analysis also documents the disappearance of the disease from the United Kingdom during the twentieth century, which appears to have been an unintentional result of the introduction of dog control policies.

## Background

**Figure 1:**
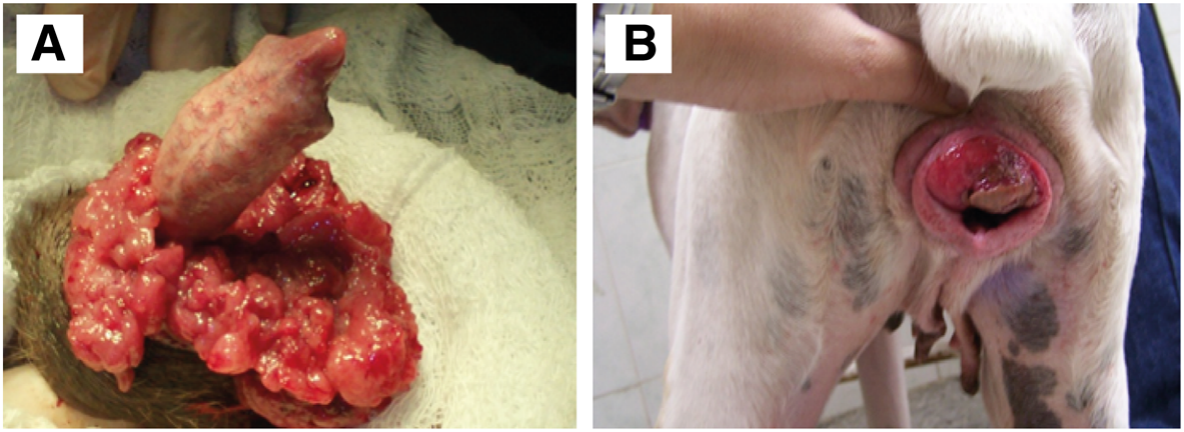
**Canine transmissible venereal tumour (CTVT).** CTVT affecting **(A)** a male dog **(B)** a female dog.

The canine transmissible venereal tumour (CTVT) is a naturally occurring transmissible cancer which is spread between dogs by the allogeneic transfer of living cancer cells. The disease is usually transmitted during coitus [1]-[6] and results in the appearance of tumours most often associated with the external genitalia of male and female dogs (Figure 1). CTVT has been reported in many countries around the world (reviewed in [7]-[12]) and is, to our knowledge, the oldest and most prolific mammalian somatic cell lineage [13]-[15].

Genetic evidence suggests that CTVT first arose from the somatic cells of an individual dog that lived several thousand years ago [13]-[15]. Despite its ancient origin, global populations of CTVT diverged only within the last few hundred years [13]-[15], suggesting that CTVT spread around the globe relatively recently. Despite numerous historical and contemporary reports of the disease (reviewed in [7]-[12],[16]), no systematic study of CTVT’s distribution and prevalence has been performed. Estimating the worldwide distribution and prevalence of a common animal pathogen such as CTVT is a challenging task [17]-[22]; in most countries the disease is not considered notifiable, and in many areas animals do not have access to veterinary care. Veterinary records are often scant or inconsistent, and variation in CTVT prevalence may exist within countries due to seasonal, demographic or geographic factors. Here, we used a crowdsourcing approach to estimate CTVT’s distribution and prevalence by using the internet to contact and source local CTVT prevalence estimates from 645 veterinarians and animal health workers worldwide. We show that the disease’s prevalence is linked to national development status and that dog management policies leading to declines in the population of free-roaming dogs may have caused its recent eradication from the United Kingdom. Further insight into the epidemiology of CTVT may help elucidate the timing, route and manner of the disease’s global spread. In addition, an understanding of the factors influencing CTVT distribution and prevalence may inform policy decisions for CTVT control.

## Results

### Historical worldwide CTVT distribution

We initially analysed the published literature in order to understand the historical distribution of CTVT. We found 317 reports of primary naturally occurring CTVT cases in the published literature (see Additional file 1), including case reports, experimental reports and retrospective studies. These records provide evidence of CTVT on all six inhabited continents and range in date from 1810 until the present time (see Additional file 1 and Additional file 2). The earliest known record of CTVT is from London in 1810, where it was noted as one of only two cancers known to afflict dogs [23]. We found evidence that CTVT was present prior to 1910 in the United States [24],[25], France [26], Germany [27]-[33], Italy [34], the United Kingdom [2],[4],[6],[35]-[37], Japan [38] and Papua New Guinea [39]. A 1906 report from Papua New Guinea stated that CTVT was “endemic before the advent of the white man” [39]. Two reports published in the 1950s noted declines in CTVT prevalence, stating that CTVT occurs “less commonly in London dogs” (1954) [40] and that there has been a “reduction in incidence” of CTVT in New York City (1951) [41]. Indeed, our literature review documented a clear decline in CTVT in the United Kingdom during the twentieth century (see Additional file 3).

Eighteen of the published reports provided a numerical value for the prevalence of naturally occurring CTVT in their study (see Additional file 4). The reported prevalence of CTVT in affected populations ranged from 1% or less (Jamaica, 1968 [42]; Kenya, 1972 [43]; Bangladesh, 2010 [44]) to almost 20% (Papua New Guinea, 1985 [45] and 1986 [46]; Mexico 2007 [47] and 2010 [48]).

### Current worldwide CTVT distribution

We designed and distributed an internet-based questionnaire with 18 predominantly multiple choice questions regarding CTVT prevalence, features of CTVT-infected dogs and conditions of local dog populations to more than one thousand veterinarians and animal health workers in 164 countries (full questionnaire is available in Additional file 5). Respondents were asked to estimate the prevalence of CTVT in their local area. We received a total of 645 completed questionnaires from 109 countries.

**Figure 2:**
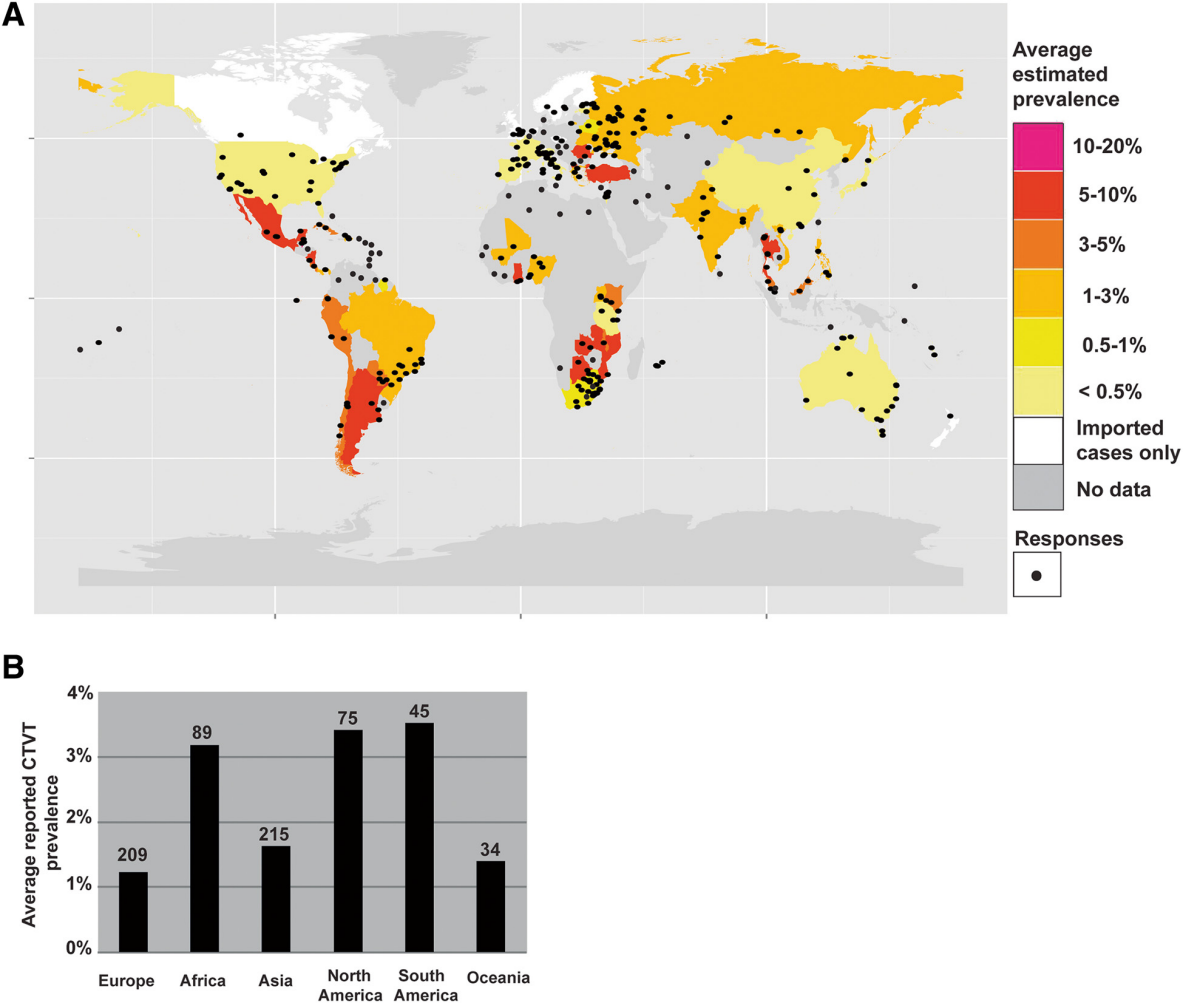
**Worldwide CTVT distribution and prevalence. (A)** CTVT worldwide distribution by country. The colour of each country represents the average of all CTVT prevalence estimates obtained from that country. The location of each response is indicated with a black dot. Countries from which fewer than three responses were received are coloured in grey. Complete datasets are included in Additional file 6 and Additional file 7. Map was created using the *maptools* and *ggmap* packages, implemented in R. **(B)** Average reported CTVT prevalence by continent. The number of responses estimating prevalence in each continent is shown above each bar.

The average estimated CTVT prevalence reported for each country from which a minimum of three responses were received is displayed in Figure 2A (see also Additional file 6). In addition, a separate map displays each individual respondent’s estimate of CTVT prevalence (see Additional file 7). The data indicate that CTVT is estimated to occur at between one and ten percent prevalence in dogs in most countries in South and Central America as well as in parts of Africa and Asia. The average reported prevalence by continent is shown in Figure 2B. The highest estimated CTVT prevalence that we recorded was in Belize, where the average CTVT prevalence (calculated from 6 responses) was estimated to be between 10 and 20 percent. Several countries (Canada, the Czech Republic, Finland, the Netherlands, New Zealand, Sweden, Switzerland and the United Kingdom) were consistently reported by all respondents from that country to be free of endemic CTVT; in these countries, the only CTVT cases were specifically reported to be imported from abroad (see also Figure 2A and Additional file 7). CTVT was reported as absent from many regions of the United States and Australia, but was present in remote indigenous communities, including Indian reservations in Arizona and North Dakota, as well as in Australian Aboriginal communities in the Northern Territory and Western Australia (see Additional file 7). There was also geographical variation in estimated CTVT prevalence in Europe; the disease was reported to be absent except for occasional imported cases in many countries of Northern and Western Europe, but was estimated to be present at less than ten percent prevalence in countries in Southern and Eastern Europe (see Additional file 7).

### Status of dogs with CTVT

**Figure 3:**
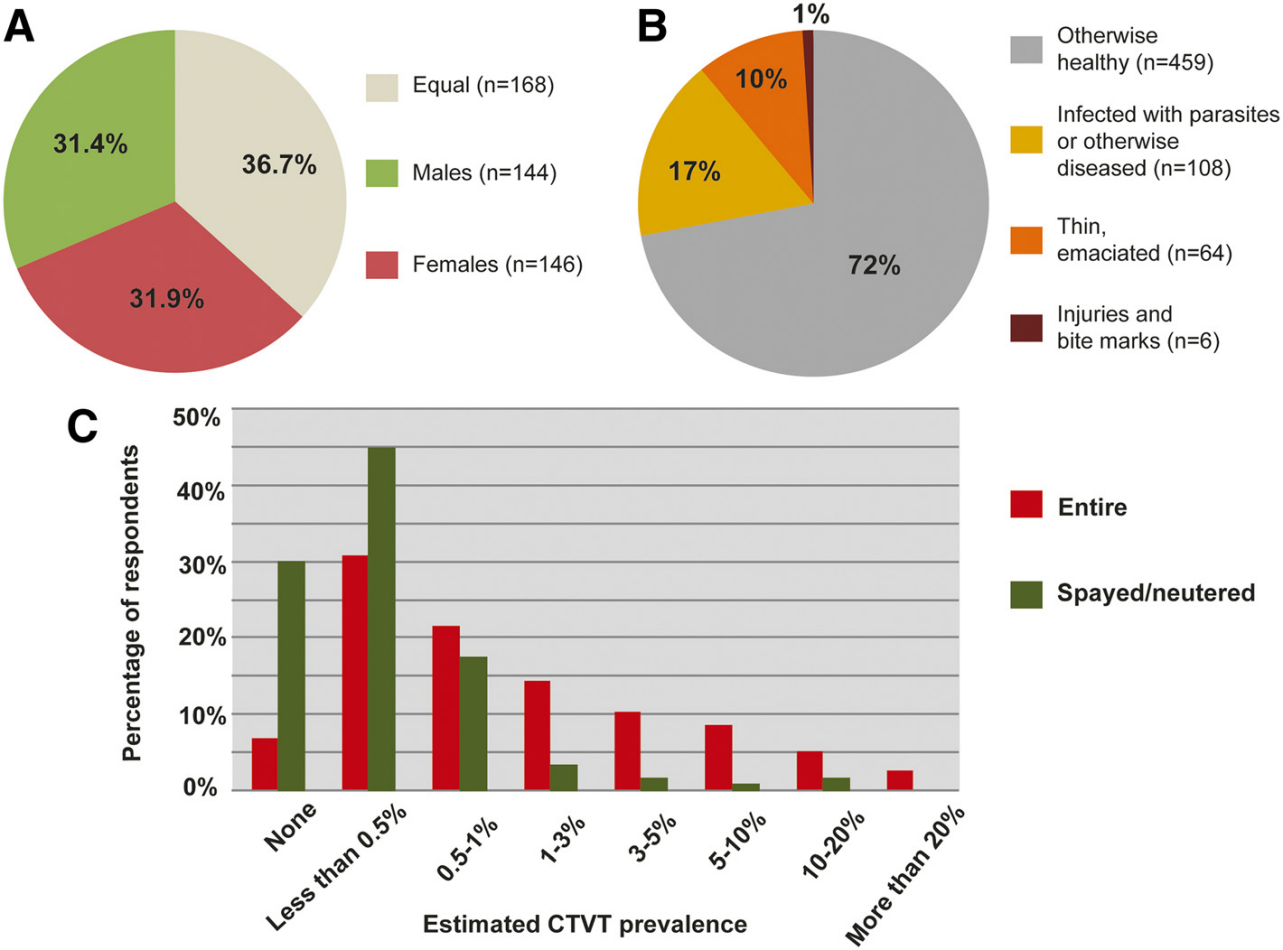
**Status of dogs with CTVT. (A)** Gender of dogs infected with CTVT. Respondents were asked if they observed CTVT “more commonly in males”, “more commonly in females” or “equally in males and females”. The numbers refer to the number of respondents choosing each option. **(B)** Health condition of dogs infected with CTVT. Respondents were asked to report on the condition of the majority of CTVT-infected dogs by selecting one of the four categories shown. The numbers refer to the number of respondents choosing each option. **(C)** Relationship between spaying/neutering and CTVT prevalence. CTVT prevalence estimates from respondents who claimed that the majority of dogs in their area were spayed/neutered (total of 120 respondents) or entire (total of 340 respondents) are coloured in red and green respectively. Percentage of respondents refers to the proportion of respondents choosing each option.

We asked questionnaire respondents to report the most likely gender and health status of animals with CTVT. Despite previous suggestions that CTVT is more common in males [49],[50] or females [51]-[55], responses to the questionnaire indicated that there is no universal gender bias for CTVT infection, with 144 respondents reporting that CTVT was more common in males, 146 reporting that it was more common in females and 168 claiming that it was equally common in both sexes (chi-squared test for no gender difference, p = 0.907) (Figure 3A). The majority of respondents (459 out of 637) claimed that dogs with CTVT were otherwise healthy rather than infected with parasites or otherwise diseased, thin or emaciated, or carrying injuries or bite marks (Figure 3B). We investigated the association of spaying/neutering with CTVT presence by asking respondents whether the majority of dogs in their country are spayed/neutered or entire. The responses showed that higher estimated CTVT prevalence coincides with higher percentage of respondents claiming that the majority of dogs are entire rather than spayed or neutered (Figure 3C).

Despite previous reports of successful experimental transmission of CTVT into wild canids, including wolves, coyotes and red foxes [3],[30],[56]-[58], no naturally occurring CTVT case has been previously reported in a wild canid. We further investigated this by asking all questionnaire respondents if they had observed CTVT in a wild canid (see Additional file 5). The responses did not reveal any confirmed reports of CTVT in wild canid populations.

### Factors influencing CTVT prevalence

**Figure 4:**
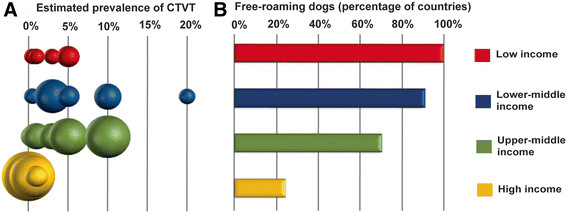
**Factors associated with CTVT prevalence. (A)** Relationship between CTVT prevalence and socio-economic status defined as low/lower-middle/upper-middle/high income economies (World Bank, Country and Lending Groups [59]). The size of the dots represents the number of countries with estimated average CTVT prevalence within each interval. CTVT prevalence values represent the upper limit of each categorical interval. **(B)** Relationship between socio-economic status defined by division into low/lower-middle/upper-middle/high income economies and presence of free-roaming dogs, as reported by respondents to the questionnaire. “Percentage of countries” refers to the proportion of countries within each income category in which the presence of free-roaming dogs was reported by the majority of respondents.

We next stratified countries based on income economy (The World Bank, Country and Lending Groups [59]) and found strikingly lower estimated prevalence of CTVT in countries with high income economies, compared with those with low, lower-middle and upper-middle income economies, which had similar distributions of estimated CTVT prevalence (Figure 4A). The proportion of countries with reported presence of free-roaming dogs was also associated with income economy category (Figure 4B), presenting the possibility that differences in presence of free-roaming dogs may explain the link between CTVT prevalence and national development status.

Additionally, we observed a weak negative correlation between average reported CTVT prevalence and socio-economic status, determined by Gross Domestic Product (GDP) per capita value for each country (see Additional file 8, Pearson’s correlation two tailed test between CTVT prevalence and GDP values, r = −0.504, p = 4.52 × 10^−5^), together with a weak negative correlation between average reported CTVT prevalence and distance from the equator, as measured by distance from the equator to the capital city of each country (see Additional file 8, Pearson’s correlation two tailed test between CTVT prevalence and distance from the equator, r = −0.416, p = 8.39 × 10^−4^). It is possible, however, that these correlations, as well as a previously identified correlation between CTVT prevalence and distance from the equator within the United States [60], may be explained by the presence of free-roaming dogs.

### Metastasis and treatment of CTVT

**Figure 5:**
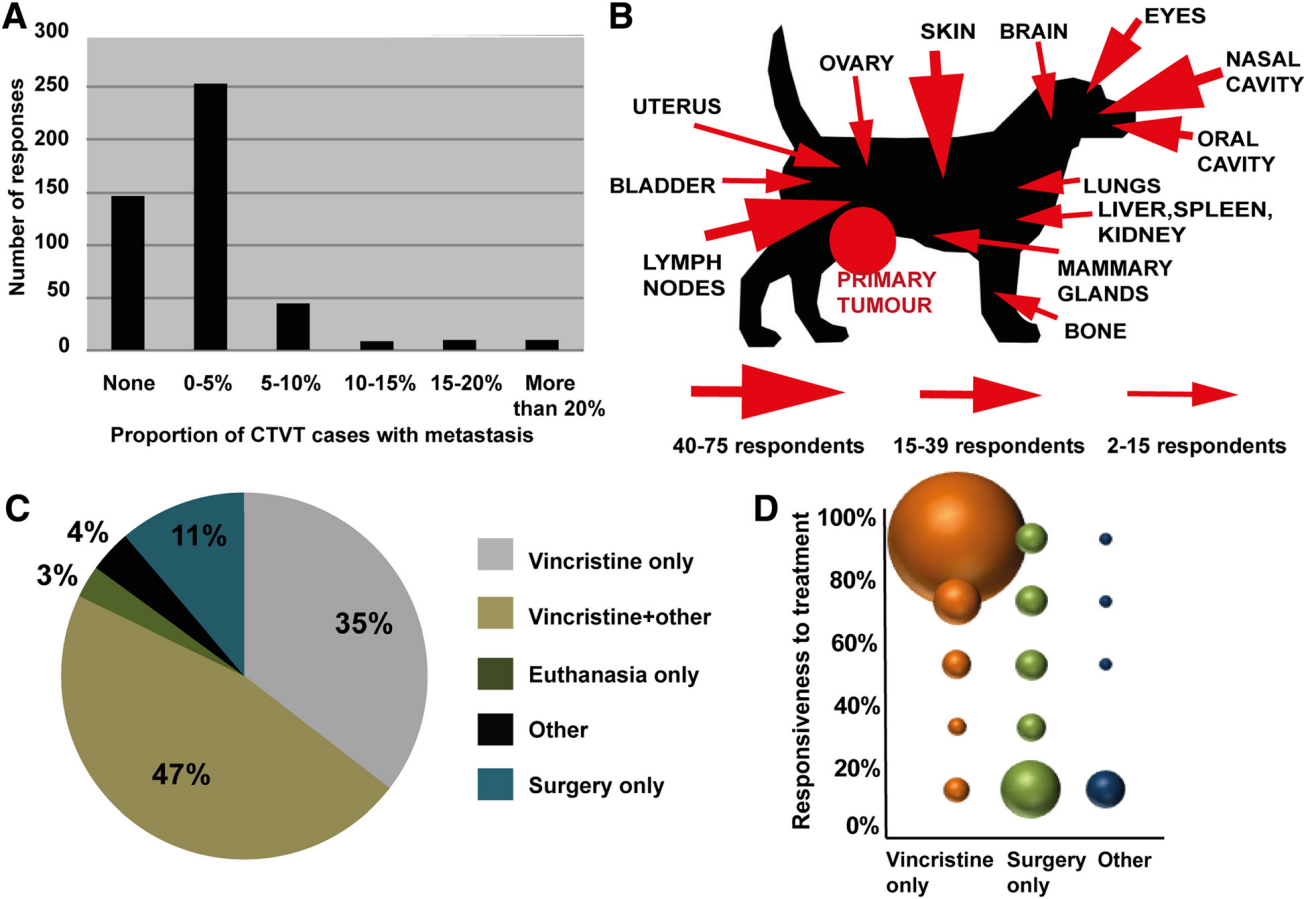
**CTVT metastasis and treatment. (A)** Estimated proportion of CTVT cases with metastasis. Number of responses refers to the number of respondents choosing each option. **(B)** Sites of CTVT metastasis reported by respondents. The size of the arrow is proportional to the number of respondents who reported observations of CTVT metastasis at this site. **(C)** CTVT treatments reported by respondents. Total number of responses for this question was 454. **(D)** CTVT response to treatment. “Response to treatment” refers to the proportion of CTVT cases that respondents estimated went into complete remission after the selected treatment. “Other” treatments included euthanasia, aloe vera treatment and electrocautery, but do not include chemotherapy.

CTVT metastasis has been previously noted in individual case reports [43],[50],[52],[54],[61]-[86]. We asked questionnaire respondents to estimate the proportion of CTVT cases in which metastasis is observed. Of the six categorical intervals available to respondents (“none”, “0-5%”, “5-10%”, “10-15%”, “15-20%”, “more than 20%”), the majority of respondents estimated that metastasis occurs in 0–5 percent of CTVT cases (Figure 5A), consistent with previously published estimates [1],[49]. Additionally, we recorded the most commonly observed sites of metastasis reported by questionnaire respondents (Figure 5B).

Vincristine is known to be a very effective treatment for CTVT [49],[53],[87]-[90]. We asked respondents to report the type and effectiveness of treatments they typically administer for CTVT (Figure 5C). The majority of respondents (373 out of 454 respondents, 82.2%) reported that they use either vincristine alone or vincristine in combination with surgery, doxorubicine or radiotherapy. A proportion of respondents (10.9%, 50) reported that they used surgery alone or other non-vincristine treatments (4%, 18). Thirteen (2.9%) respondents stated that the only option is euthanasia. Those who used vincristine treatment for CTVT reported that 80 to 100 percent of tumours usually went into complete remission after treatment (Figure 5D), however the number of vincristine doses claimed to be required for complete remission varied between respondents. This contrasted with the poor reported effectiveness of surgery alone or other non-vincristine treatments (Figure 5D).

## Discussion

This survey of current and historical CTVT distribution patterns confirms that CTVT was and remains a very common canine infectious disease throughout the world. The global CTVT prevalence data reported in this study are individual estimates of local CTVT prevalence and are thus subject to errors introduced by variation in methodologies used by respondents to estimate prevalence (see Methods). Difficulties in prevalence estimation may have been further confounded by a combination of absent, inconsistent or incomplete record keeping, personal biases as well as systematic biases introduced where the sampling population was not representative of the population as a whole. Furthermore, variation in CTVT prevalence due to seasonal, demographic or local geographical factors may not have been captured by our approach. In order to minimise the effects of estimate biases on our analysis, we only included data from countries from which we received at least three responses in Figure 2A and Figure 4. Despite the limitations associated with estimating global CTVT prevalence, our large sample size supports the conclusion that a strikingly large proportion of the global dog population harbours CTVT infection at a prevalence of between 0.5 and 10 percent. Future studies will be important to further validate global variation in CTVT prevalence.

**Figure 6:**
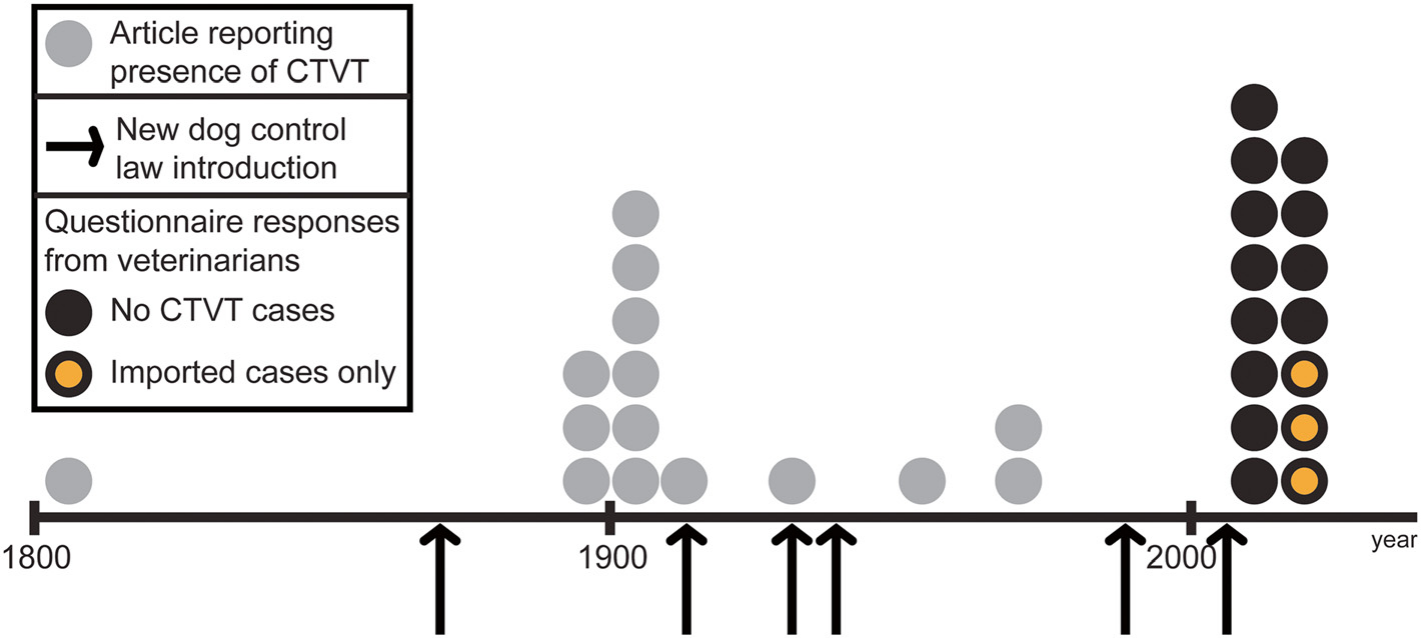
**Disappearance of CTVT from the United Kingdom during the twentieth century.** Timeline showing the declining number of historical reports of CTVT in the United Kingdom, coinciding with the introduction of dog laws. Data from the questionnaire (indicated with black and yellow dots) were used to confirm current absence (except for occasional imported cases) of CTVT from the United Kingdom. See also Additional file 3.

We have documented the decline and disappearance of CTVT from the United Kingdom during the twentieth century (Figure 6 and Additional file 3). The eradication of CTVT from the United Kingdom may be due to the introduction of a series of dog management laws throughout the nineteenth and twentieth centuries (Figure 6). The Dogs Act, 1871 [91] imposed civil responsibility on dog owners and stated that dogs must be “under proper control” and “stray dogs may be detained and sold or destroyed”. This was followed by the Dogs Act 1906 [92], amended in 1928 and 1938, which introduced a requirement to report stray dogs to the police. It is striking that the eradication of CTVT, once a common canine pathogen in the United Kingdom (see Additional file 3), appears to have occurred as an unintentional result of human intervention.

The importance of dog management and spaying/neutering in CTVT control was highlighted by a respondent from Koh PhaNgan Island, Thailand, where breeding control and sterilisation campaigns have almost eradicated CTVT from the island since the commencement of the sterilisation project in 2001. Several respondents, however, when asked to comment on any unusual cases of CTVT that they had seen, commented that they had observed CTVT in dogs years after spay or neuter surgery. This suggests that either the latent period for CTVT development, which previous anecdotal reports have suggested may last for weeks or months [2],[55],[93], can sometimes last years, or, alternatively, that spaying and neutering does not always protect dogs from CTVT. Furthermore, non-coital modes of CTVT transmission, including biting, licking or sniffing, may also contribute to CTVT infection of spayed/neutered dogs [68],[94]-[98].

Despite the widespread presence of CTVT in dog populations worldwide, the results of our survey indicate that its prevalence rarely rises above 10% (Figure 2). This contrasts with epidemiological patterns observed for the only other known naturally occurring transmissible cancer, the Tasmanian devil facial tumour disease (DFTD). Prevalence of DFTD usually rises above 50% in affected Tasmanian devil populations and the disease usually triggers a rapid population decline (reviewed in [99]). Given that the mixed mating system of dogs would promote widespread exposure to the disease [100], this pattern suggests that only a proportion of the dog population may be susceptible to CTVT at any one time and, possibly, as one early report of CTVT proposed, that “some animals are naturally refractory” to infection [5]. Future studies of CTVT exposure and susceptibility in free-roaming dog populations may reveal further insights into the biological basis of this interesting observation.

Genetic studies indicate that the global spread of CTVT has occurred relatively recently in the history of the lineage, probably within the last 500 years [13]-[15]. Although we do not know the location in which CTVT first emerged, our study has highlighted the remarkable efficiency with which CTVT has colonised its global host population. We obtained evidence for CTVT’s presence in some of the world’s most isolated communities and islands, including the Solomon Islands, Samoa, American Samoa, Fiji, Reunion, Mauritius and several islands in Micronesia. In contrast, New Zealand is free of CTVT, likely due to its rigorous import quarantine rules [101]. Together, these findings highlight the mobility of dogs, which, alongside humans, have travelled rapidly and extensively around the globe.

This study has provided information on historical and current CTVT global distribution and prevalence and has illuminated a number of factors which may influence CTVT prevalence, including presence of free-roaming dogs, dog spay/neuter practices and enforcement of dog control laws. In addition to providing insight into the global spread of a unique type of pathogen, this study may assist policy-makers and veterinarians in the development of measures to more effectively control and reduce CTVT prevalence and prevent further spread of the disease.

## Conclusions

Our results provide a survey of the current global distribution of CTVT, confirming that CTVT is endemic in at least 90 countries worldwide. Additionally, our analysis highlighted factors that continue to modify CTVT’s prevalence around the world; free-roaming dogs were implicated as a reservoir for the disease and spaying and neutering were associated with lower CTVT prevalence. Our analysis also documents the disappearance of the disease from the United Kingdom during the twentieth century, which may have been an unintentional result of the introduction of dog control policies. In addition to providing insights into the global spread of a unique canine infectious disease, this study may assist veterinarians and policy-makers to develop measures to more effectively control and reduce CTVT prevalence and spread.

## Methods

This study was approved by the Department of Veterinary Medicine, University of Cambridge, Ethics and Welfare Committee (reference number CR105). The questionnaire used in this study is available in supplementary materials (see Additional file 5). Potential participants were selected with an internet-based search. The questionnaire was sent by email to 1025 individuals and distributed at several veterinary conferences. The questionnaire was also circulated in several veterinary societies’ mailing lists and published in veterinary periodicals, newsletters and on social media sites. Of the 645 completed questionnaires received, nine respondents provided information about more than one country. Most of the respondents were private veterinarians (415, 64.3%) or veterinarians working at veterinary schools (143, 22.2%), but they also included other individuals working at charitable organisations (50, 7.8%), government (16, 2.5%), pathology laboratories (13, 2.0%) and research agencies (8, 1.2%). All of the respondents completed the questionnaire within days or weeks of receiving it. The questionnaire was made available in English, Chinese, French, Portuguese, Spanish and Russian.

The estimated average CTVT prevalence for each country was determined using the average of the mid-point values for each reported prevalence category received from each country. Only countries with three or more responses were included in the analyses in Figures 2A and 4, but all responses are shown in Additional file 6 and Additional file 7. Previous studies indicated that CTVT prevalence rarely rises above 20 percent (see Additional file 4), therefore the prevalence categories available in the questionnaire were “none”, “less than 0.5%”, “0.5-1%”, “1-3%”, “3-5%”, “5-10%”, “10-20%” and “more than 20%”. The CTVT prevalence figures reported here were estimated by respondents based on (1) an estimate of the number of CTVT patients as a proportion of total canine patients in general clinical practice; (2) an estimate of the total number of CTVT diagnostic samples as a proportion of the total number of canine diagnostic samples passing through a pathology laboratory; (3) a personal estimate based on previous veterinary experience and discussions with veterinary colleagues; and (4) the total number of CTVT cases in populations of dogs participating in spay/neuter campaigns.

The World Atlas [102] was used to generate the list of countries in Additional file 6, and Taiwan and Reunion were added as separate countries. Data on the distance from the equator and GDP values were obtained from News Track India [103] and the International Monetary Fund [104] respectively. Distance from the equator for each country refers to the distance of the capital city from the equator. Countries were classified as low income, lower-middle income, upper-middle income and high income economies based on the classification scheme defined by the World Bank [59].

## Competing interests

The authors declare that they have no competing interests.

## Authors’ contributions

AS conceived and designed the study, performed the study, analyzed the data and wrote the paper. EPM conceived and designed the study, performed the study, wrote the paper and acted as the first author’s study supervisor. Both authors read and approved the final manuscript.

## Additional files

## Supplementary Material

Additional file 1 **Contemporary and historical reports of naturally occurring CTVT cases in the published literature.** Reports are ordered by date. Multiple reports from the same publication are indicated on separate lines. Only reports referring to primary naturally occurring CTVT cases are included, while articles reporting only cases of experimentally transplanted CTVT are excluded. Click here for file

Additional file 2 **Global distribution of published reports of CTVT.** Locations in which naturally occurring CTVT cases are reported in the published literature are indicated on the map, classified by date of report. Bibliographical information for each case is found in Additional file 1. The two reports in Canada [5],[105] were specified as imported cases from abroad and are marked with *. Map was created using Adobe Illustrator. Click here for file

Additional file 3 **Historical reports confirming presence of CTVT in the United Kingdom in the 19th and 20th centuries.** The comments were obtained from articles published between 1810 and 1969. Click here for file

Additional file 4 Contemporary and historical reports providing a numerical value for CTVT prevalence. Click here for file

Additional file 5 Text version of CTVT questionnaire used in this study. Click here for file

Additional file 6 Summary of CTVT prevalence data by country. Click here for file

Additional file 7 **Worldwide CTVT distribution and prevalence.(A)** Map indicating CTVT prevalence estimated by each respondent. Each response is represented by a single coloured dot. Map was created using Adobe Illustrator. **(B)** Higher magnification map showing detailed distribution of CTVT prevalence estimates in Europe. Map was created using Adobe Illustrator. Click here for file

Additional file 8 **Socio-economic and climatic factors associated with CTVT prevalence. (A)** Relationship between estimated CTVT prevalence and Gross Domestic Product (GDP) per capita values in US dollars. Prevalence values displayed represent the higher limit of each categorical interval. Each dot represents the average of three or more estimated prevalence values received from that country. A line of best fit is displayed (R^2^ = 0.2543). **(B)** Relationship between estimated CTVT prevalence and geographical latitude displayed as distance from the equator (in degrees of latitude) measured for the capital city of each country. Prevalence values displayed represent the higher limit of each categorical interval. Each dot represents the average of three or more estimated prevalence values received from that country. A line of best fit is displayed (R^2^ = 0.1727). Click here for file
