# Supplementary material for: The changing global distribution and prevalence of canine transmissible venereal tumour
Source: BMC Vet Res. 2014 Sep 3;10:168. doi: 10.1186/s12917-014-0168-9 (PMC4152766; doi:10.1186/s12917-014-0168-9)
Supplement: Additional file 1 — Contemporary and historical reports of naturally occurring CTVT cases in the published literature. Reports are ordered by date. Multiple reports from the same publication are indicated on separate lines. Only reports referring to primary naturally occurring CTVT cases are included, while articles reporting only cases of experimentally transplanted CTVT are excluded. [file s12917-014-0168-9-S1.pdf]

**Additional file 1. Contemporary and historical reports of naturally occurring CTVT cases in the published literature.** Reports are ordered by date. Multiple reports from the same publication are indicated on separate lines. Only reports referring to primary naturally occurring cases are included, while articles reporting only cases of experimental transplantation are excluded.

| Year | Location | First Author | Title | Reference |
|---|---|---|---|---|
| 1810 | UK (London) | D. P. Blaine | A domestic treatise on the diseases of horses and dogs | Published 1810, 4th edition, 161-2, 1810 |
| 1876 | Russia (St.Petersburg) | M. A. Nowinsky | Zur Frage uber die Impfung der krebsigen Geschwulste | Zbl Med Wissench 14, 790-791, 1876 |
| 1888 | Germany (Lemberg) | Wehr | Demonstration der durch Impfung von Hund auf Hund erzeugten Carcinomknotchen | Zbl Chir 15 (suppl. to no. 24), 8-9, 1888 |
| 1889 | Germany (Lemberg) | Wehr | Weiter Mittheilungen uber die positiven Ergebnisse der Carcinom-Ueberimpfungen von Hund auf Hund | Arch Klin Chir 39, 226-228, 1889 |
| 1894 | France (Paris) | S. Duplay | Tumeurs expérimentales chez les animaux | Atti dell'XI Congresso Medico Internazionale, Roma, 2, 103-104, 1894 |
| 1895 | Germany (Berlin, Gottingen) | E. von Bergmann | Gelungene Carcinomubertragung beim Hunde | Zbl Chir 27, XXIV. Kongress, 1895 |
| 1895 | Poland (Wroclaw) | E. von Bergmann | Gelungene Carcinomubertragung beim Hunde | Zbl Chir 27, XXIV. Kongress, 1895 |
| 1897 | UK (London) | G. B. Smith | Infective venereal tumours in dogs | Transactions Pathological Society London, 48, 310-323, 1897 |
| 1897 | UK (London) | Pathological society of London | Meeting held on 6th April 1897 | The Lancet, April 10, 1025-1026, 1897 |
| 1898 | UK (London) | G. B. Smith | Infective venereal tumours in dogs | J Pathol Bacteriol 5, 99-110, 1898 |
| 1900 | UK (London) | F. Hobday | Operations on the genital organs | In Canine and Feline Surgery, W.&A.K. Johnston, 1900 |
| 1902 | UK (Leeds) | C. Powell White | Contagious growths in dogs | Br Med J July 19, 2, 176-177, 1902 |
| 1904 | Germany (Frankfurt) | A. Sticker | Transplantables Lyphosarkom des Hundes | Z Krebsforsch 1, 413-444, 1904 |
| 1904 | Papua New Guinea (Port Moresby) | C. G. Seligmann | On the occurrence of new growths among the natives of British New Guinea | Third Scientific Report Invest Imp Cancer Res Fund, 26-40, 1908 |
| 1905 | UK (London) | F. Hobday | Observations on Contagious Venereal Tumours in Canine Patients | Vet J 2 (new series), 342-346, 1905 |
| 1905 | UK (London) | E.F. Bashford | Comparison between the transmission of an infective granuloma of the dog and carcinoma of the mouse | Scientific report, Imp Cancer Res Fund 2, 33-37, 1905 |
| 1906 | Canada (Montreal) - imported from the UK | C. French | Surgical diseases and surgery of the dog | Published by Washington D.C. pp. 254-256, 286-287 and 365-367 |
| 1906 | UK (London) | F. Hobday | Tumours on the Penis and Prepuce, Malignant Tumours of the Vagina | Surgical diseases of the dog and cat and anaesthetics, second edition, Bailliere, Tindall and Cox, 8 Henrietta Street, 1906 |
| 1906 | Germany (Berlin) | A. Sticker | Ubertragung von Tumoren bei Hunden durch den Geschlechtsakt | Berl Tier Woschr 50, 894-995, 1906 |

| | | | | |
|---|---|---|---|---|
| 1906 | US (New York City) | S.P. Beebe | A study of the so called infectious lymphosarcoma of dogs | J Med Res 15, 209-227, 1906 |
| 1907 | US (New York City) | S.P.Beebe | The growth of lymphosarcoma in dogs-summary of recent observations | J Am Med Assoc 49, 18, 1492-1493, 1907 |
| 1907 | Germany (Berlin) | P. Bergell (A. Sticker) | Ueber Pathogenese und uber den spezifischen Abbau der Krebsgeschwulste | Deut Med Wochenschr 2, 38, 1521-1522, 1907 |
| 1907 | France (Paris) | M.A. Borrel | Lymhosarcome du chien | Sem Med (Paris) 27, 94-95, 1907 |
| 1907 | France | M. Cadéac | Infection sarcomateuse aigue du chien | J Med Vet Zootech 58, 266-269, 1907 |
| 1907 | UK (Edinburgh, London) | H. Wade | An experimental investigation of infective sarcoma of the dog, with a consideration of its relationship to cancer | J Pathol 12, 384-425, 1907 |
| 1907 | Germany (Berlin) | A. Sticker | Endemischer Krebs | Z Krebsforsch 5, 2, 215-224, 1907 |
| 1908 | Sri Lanka | C. G. Seligmann | On the occurrence of new growths among the natives of British New Guinea | Third Scientific Report Invest Imp Cancer Res Fund, 26-40, 1908 |
| 1909 | Japan | Y. Matsui | Uber transplantable-sarcomatige Neubildung des Hundes | Gann 4, 123, 1909 |
| 1921 | US (New York City) | W. L. Williams | the Venereal Tumors of the Dog. Venereal Granulomata. Lympho-sarcoma. | In The Diseases of the Genital Organs of Domestic Animals, published by Ithaca, N.Y., 1921 |
| 1927 | Japan | S. Matsuba | Studies on venereal tumor in dog | J Jap Soc Vet Sc 6, 167, 1927 |
| 1929 | US (Minnesota, Rochester) | W. H. Feldman | So called infectious sarcoma of the dog in an unusual anatomic situation | Am J Path 5, 183-195, 1929 |
| 1931 | Germany (Berlin) | H. Auler | Uber Tumoren des Hundes | Z Krebsforsch 35, 1-11, 1931 |
| 1932 | US (Minnesota, Rochester) | W. H. Feldman | Transmissible Lymphosarcoma of Dogs | In Neoplasms of Domesticated Animals, Chp 20, 343-56, Philadelphia, WB Saunders Co., 1932 |
| 1932 | UK | W. H. Feldman | Transmissible Lymphosarcoma of Dogs | In Neoplasms of Domesticated Animals, Chp 20, 343-56, Philadelphia, WB Saunders Co., 1932 |
| 1932 | China (Beijing) | A. I. H. Wong | Radium treatment in the so-called infectious sarcoma of dogs | Chin Med J-Peking 46, 377-382, 1932 |
| 1933 | US (Tennessee, Nashville) | W. A. DeMonbreun | An experimental investigation concerning the nature of contagious lymphosarcoma of dogs | Am J Cancer 21, 295-321, 1933 |
| 1933 | US (Pennsylvania, Philadelphia) | E. L. Stubbs | Experimental studies on venereal sarcoma of the dog | Am J Pathol 10, 275-286, 1933 |
| 1934 | France (Lyon, Collonges, Mureils) | R. Bauduin | Les Papillomes du vagin chez la chienne | Thesis presented at La Faculte de Medicine et de Pharmacie de Lyon, 30th January 1934, Editors: BOSC Freres, M&L RIOU, 1934 |
| 1938 | France (Toulouse) | R. Lasserre | Recherches sur le Cancer des animaux domestiques | Rev Med Vet 90, 425-451, 1938 |
| 1938 | Italy (Bologna) | F. Cella | Spleno-reticolo-sarcoma e sarcoma di Sticker in un cane | La Nuova Veterinaria 304-309, 1938 |

| | | | | |
|---|---|---|---|---|
| 1939 | Italy (Messina) | P. Ajello | Osservazioni sul tumore genitale contagioso del cane | Thesis presented at Facolta do medicina veterinaria della R. Universita di Messina, 1939 |
| 1939 | New Guinea | P. Ajello | Osservazioni sul tumore genitale contagioso del cane | Thesis presented at Facolta do medicina veterinaria della R. Universita di Messina, 1939 |
| 1939 | France (Paris) | L. Bory | Le lympho-sarcome infectieux des Chiens | Clinique et Pathologie comparee, Paris, Masson, 23-25, 1939 |
| 1939 | Italy (Bologna) | F. Cella | Sopra un secondo caso di granuloma venereo del cane (c.d. sarcoma di sticker) osservato nell'Emilia - metastasi splenica | La Nuova Veterinaria 12, 217-220, 1939 |
| 1944 | South Africa (Pretoria) | C. Jackson | The Cytology of the Contagious (Venereal) Tumour of the Dog | Onderstepoort J Vet Sci and Anim Indus 20, 97-118, 1944 |
| 1946 | France | G. Lesbouyries | Néoplasies vénériennes du Chien et de la Chienne | Recueil Med Vet 122, 1, 7-19, 1946 |
| 1947 | Italy (Sardinia) | A. Carta | Indagini sperimentali sul cosi detto sarcoma di sticker | Profilassi 20, 22-23, 1947 |
| 1947 | Ireland (Waterford) | L. N. Gleeson | An Account of Infectious Venereal Granuloma in the Greyhound | Vet Rec 59, 411-413, 1947 |
| 1949 | Puerto Rico (San Juan) | J. H. Rust | Transmissible Lymphosarcoma in the Dog | JAVMA 114, 10-14, 1949 |
| 1949 | France (Toulouse) | F. Gouaud | Contribution a l'etude du sarcome de Sticker: therapeutique par les rayons X | Thesis presented at the Veterinary School in Toulouse in 1949 |
| 1949 | France (Toulouse) | P. Lacour | Contribution a l'etude du sarcome de sticker: etude clinique, cytologique et therapeutique basee sur 66 observations originales: conception | Thesis presented at the Veterinary School in Toulouse in 1949 |
| 1949 | France (Toulouse) | M. Neveu | Contribution a l'etude du 'Sarcome de Sticker' essai de therapeutique, deductions histo-physiologiques | Thesis presented at the Veterinary School in Toulouse, 1949 |
| 1950 | France (Lyon) | P.Collet | Essais de traitement des tumeurs vénériennes du chien par la podophylline (sarcome de Sticker) | Bull Soc Sci Vet Lyon, 52, 115-122, 1950 |
| 1950 | France (Toulouse) | I. Nanta | Les tumeurs veneriennes du chien. Cadre nosologique. | Rev Vet 101, 298-320, 356-366, 1950 |
| 1950 | France (Toulouse) | P. Genty | Contribution a l'etude du Sarcome de Sticker. Epidemiologie de l'affection | Thesis presented at the Veterinary School in Toulouse, 1950 |
| 1950 | France (Toulouse) | J. P. Salacroup | Contribution a l'etude du sarcome de sticker: consideration etiologique de l'affection | Thesis presented at the Veterinary School in Toulouse, 1950 |
| 1951 | US (New York) | F. Bloom | The Transmissible Venereal Tumour of the Dog. Studies Indicating That the Tumor cells are Mature End Cells of Reticulo-endothelial Origin | Am J Path 27, 119-139, 1951 |
| 1952 | Mali (previously French Sudan) (Bamako) | Z. Derbal | Traitement chirurgical des tumeurs vénériennes de la Chienne | Rec Med Vet 128, 26-29, 1952 |
| 1952 | US (Minnesota, Rochester) | A. G. Karlson | The Transmissible Venereal Tumor of Dogs: Observations on Forty Generations of Experimental Transfers | Ann NY Acad Sci 54, 1197-1213, 1952 |
| 1953 | Senegal (Dakar) | R. Jean | Action de sérum de Bogomoletz sur un sarcome de Sticker, avec métastases malignes mammaires, chez une chienne | Rev Vet Milit, 8, 93-96, 1953 |

| | | | | |
|---|---|---|---|---|
| 1953 | France (Toulouse) | J. Cadeillan | Contribution a l'etude des tumeurs veneriennes d u chien | Thesis presented at the Veterinary School in Toulouse in 1953 |
| 1954 | UK (London) | E. Cotchin | Neoplasia in the dog | Vet Rec, December 25th, Twelfth Congress paper, 879-885, 1954 |
| 1954 | US (New York City and Philadelphia) | F. Bloom | Pathology of the dog and cat, the genito-urinary system, with clinical considerations | Commonwealth Bureau of Animal Health, 275-280, 1954 |
| 1954 | France (Toulouse) | H. Parot | Contribution a l'etude des tumeurs veneriennes du chien (formes atypiques) | Thesis presented at the Veterinary School in Toulouse, 1954 |
| 1956 | Italy (Bologna) | L. Bignozzi | L'attuale valutazione istopatologica del tumore di Sticker | Veterinaria Milano, Universita di Bologna 5, 96-101, 1956 |
| 1957 | Poland (Wroclaw) | J. Utzig | Wplyw trojterpenow zswartych w zagwi brzozoweg Pliporus betulinus na guzy Stickera | Med Weter 8, 481-484, 1957 |
| 1958 | Japan (Tokyo) | M. Hataya | Effects of x-ray irradiation on the transmissible venereal tumor of the dog | Gann 49, 307-318, 1958 |
| 1958 | Japan | Y. Shirasu | Studies on the Transmissible Venereal Tumor of the Dog. Serial Transplantation into the Cheek Pouch of Hamsters. | Gann 49 (suppl.), 205-206, 1958 |
| 1958 | Japan (Hokkaido) | S. Takayama | Existence of a stem-cell lineage in an infectious venereal tumor of the dog, | Jpn J Genet 33, 56-64, 1958 |
| 1959 | US (Missouri, St.Louis) | P. V. Belkin | Extragenital Venereal Granuloma in the Abdominal Organs of a Dog | JAVMA 135, 575-576, 1959 |
| 1960 | Italy (Messina) | A. Bonaduce | Alcune Ricerche sul Tumore di Sticker | Zooprofilass 15, 937-957, 1960 |
| 1960 | Italy (Messina) | P. Ajello | Trasmissione del Tumore di Sticker con Materiale Acelurare | La Nuova Veterinaria 36, 179-183, 1960 |
| 1960 | Italy (Perugia) | G. Dozza | Anticorpi Emoagglutinanti in Cani Portatori del Cosiddetto Tumore di Sticker | Atti Soc Ital Sc Vet, 14, p 531-535, 1960 |
| 1960 | Brazil (Belo Horizonte) | L. Lazzer | Tratamiento quirurgico de tumor veneréo en perro | Rev Mil Vet 13, 140, 1960 |
| 1960 | France (Maisons-Alfort) | F. Legneau | Essai de transmission d'une néoplasie vénérienne du chien (sarcome de Sticker) | Bull Societe Francaise Dermatologie Syph 67, 542-546, 1960 |
| 1961 | Italy (Messina) | P. Ajello | Sulla Presenza di Inclusioni Nelle Cellule del Tumore di Sticker | Bull Societa Italiana Biolo Sperim 37, 247-249, 1961 |
| 1961 | Japan (Hokkaido - Sapporo, Otaru)) | S. Takayama | Cytological Studies of Tumors - A Study of Chromosomes in Venereal Tumors of the Dog | Z Krebsforsch 64, 253-261, 1961 |
| 1962 | South Africa (Cape Town) | G. de Kock | Pathological studies on neoplasms of dogs in South Africa | Onderstepoort J Vet res 29, 1, 35-54, 1962 |
| 1963 | US (Washington D.C.) | C. N. Barron | Intraocular tumours in Animals. V.Transmissible Venereal Tumor of Dogs | Am J Vet Res 24, 1263-1270, 1963 |
| 1963 | France (Paris) | J-P. Carteaud | Contribution a l'étude cytologique de la tumeur vénérienne du chien (sarcome de Sticker) | Réunion de Paris, Séance du 14 November 1963, Societe de dermatologie et de Syphiligraphie, 1963 |
| 1963 | Japan (Hyogo, Sapporo, Otaru, Osaka) | S. Makino | Some epidemiologic aspects of venereal tumors of dogs as revealed by chromosome and DNA studies | Ann NY Acad Sci 108, 1106-1122, 1963 |

| | | | | |
|---|---|---|---|---|
| 1963 | Japan (Sapporo) | T. Sofuni | A supplementary study on the chromosomes of venereal tumors of the dog | Gann 54, 149-154, 1963 |
| 1964 | US (Pennsylvania, Philadelphia) | J. E. Prier | Malignancy in a Canine Transmissible Venereal Tumor | JAVMA 145, 11, 1092-1094, 1964 |
| 1965 | India | G. A. Sastry | A case of metastatic venereal tumor in a bitch | Ind Vet J 42, 658-659, 1965 |
| 1965 | US (Pennsylvania, Philadelphia) | W. T. Weber | Chromosome Studies of a Transplanted and a Primary Canine Venereal Sarcoma | J Natl Cancer I 35, 3, 537-547, 1965 |
| 1966 | US (Alabama, Tuskegee Institute) | E. W. Adams | Canine Venereal Tumor - serum protein electrophoresis, transaminase, and lactic dehydrogenase activity | Cornell Vet 57, 572-578, 1966 |
| 1966 | Bahamas | D. A. Higgins | Observations on the Canine Transmissible Venereal Tumour as seen in the Bahamas | Vet Rec 79, 67-71, 1966 |
| 1966 | US (Alabama, Tuskegee Institute) | E. W. Adams | Growth and Maintenance of the Canine Venereal Tumor in Continuous Culture | Cancer Research 28, 753-757, 1966 |
| 1966 | France (Paris) | G. Barski | Cytogenetic Study of Sticker Venereal Sarcoma in European Dogs | J Natl Cancer I 37, 6, 787-797, 1966 |
| 1966 | US (Pennsylvania, Philadelphia) | J. M. McKenna | Some Immunological Aspects of Canine Neoplasms | Cancer Res 26, 137-142, 1966 |
| 1967 | US (Pennsylvania, Philadelphia) | R. S. Brodey | Neoplasms of the canine Uterus, Vagina and Vulva: A Clinicopathologic Survey of 90 cases | JAVMA 151, 1294-1307, 1967 |
| 1967 | France (Toulouse) | C. Lombard | Considérations sur la nature et recherches sur l'ultrastructure du sarcome de Sticker du chien | B Cancer 54, 3, 357-365, 1967 |
| 1967 | Japan (Hokkaido) | T. Koike | Successively transplanted canine transmissible sarcoma | Gann 70, 115-118, 1979 |
| 1967 | Uganda (Kampala) | O. Bwangamoi | Tumours of Domestic Animals in Uganda | Vet Rec 81, 525, 1967 |
| 1968 | Russia (Moscow) | E. S. Kakpakova | Peculiarities of the karyotype of the transmissible sarcoma cells in the dog | V Opr Onkol, 14, 43-50, 1968 |
| 1968 | Jamaica (Kingston) | M. J. Thorburn | Pathological and cytogenetic observations on the naturally occuring Canine Venereal Tumour in Jamaica (Sticker's tumour) | Brit J Cancer 22, 4, 720-727, 1968 |
| 1968 | US (Alabama, Decatur) | R. D. Powers | Immunologic Properties of Canine Transmissible Venereal Sarcoma | Am J Vet Res 29, 8, 1637-1645, 1968 |
| 1968 | India (Trichur) | P. J. Philip | Treatment of Venereal Sarcoma in a Bitch by Vulvo-Vagino-Ovario_Hysterectomy with Perineal Urethrostomy | Ind Vet J 45, 874-877, 1968 |
| 1969 | Chile (Santiago) | W. Drommer | Vergleichnende licht -und elektronenmikroskopische Untersuchungen am ubertragbaren venerischen Sarkom und Histiozytom des Hundes | Path Vet 6, 273-286, 1969 |
| 1969 | Germany (Hannover) | W. Drommer | Vergleichnende licht -und elektronenmikroskopische Untersuchungen am ubertragbaren venerischen Sarkom und Histiozytom des Hundes | Path Vet 6, 273-287, 1969 |
| 1969 | Kenya (Nairobi) | M. Murray | A Study of the Cytology and Karyotype of the Canine Transmissible Venereal Tumour | Res Vet Sci 10, 565-568, 1969 |
| 1969 | UK (London) | O.F. Jackson | Transmissible Venereal Tumour in Dogs | Vet Rec 84, 125, 1969 |
| 1969 | Ireland (Dublin) | T. D. Grimes | Transmissible Venereal Tumour in Dogs | Vet Rec 84, 124, 1969 |
| 1969 | UK (Liverpool) | J. C. Howell | Transmissible Venereal Tumour of Dogs | Vet Rec 84, 418-819, 1969 |

| | | | | |
|---|---|---|---|---|
| 1969 | Zimbabwe | J.B.Tutt | Transmissible Venereal Tumour in a Boxer Bitch | Vet Rec 84, 13, 1969 |
| 1970 | US (Alabama, Tuskegee Institute) | E. W. Adams | A Canine Venereal Tumor with Metastasis to the Brain | Path Vet 7, 498-502, 1970 |
| 1970 | US (North Carolina, Winston-Salem) | P. J. Manning | Metastasis of Canine Transmissible Venereal Tumor to the Adenohypophysis | Path Vet 7, 148-152, 1970 |
| 1970 | Hungary (Budapest) | M. Sellyei | Neue Angaben zur chromosomalen Struktur des Sticker-Sarkoms | Z Krebsforsch 74, 7-14, 1970 |
| 1970 | Uganda (Kampala) | D. H. Wright | Transmissible Venereal Sarcoma of Dogs. A Histochemical and Chromosomal Analysis of Tumours in Uganda. | Rev Europ Etudes Clin Biol 15, 155-160, 1970 |
| 1970 | Malaysia | D. H. Wright | Transmissible Venereal Sarcoma of Dogs. A Histochemical and Chromosomal Analysis of Tumours in Uganda. | Rev Europ Etudes Clin Biol 15, 155-161, 1970 |
| 1971 | US (Illinois, Chicago) | R. B. Epstein | Histocompatibility Typing and Course of Canine Venereal Tumors Transplanted into Unmodified Random Dogs | Cancer Res, 34, 788-794, 1974 |
| 1972 | Japan (Hokkaido, Ryukyu Islands) | C. G. McLeod | Transmissible Venereal Tumor with Metastases in Three Dogs | JAVMA 161, 2, 199-200, 1972 |
| 1972 | Malaysia | D. Cohen | Thymidine labelling studies in a Transmissible Venereal Tumour of the dog | Br J Cancer, 26, 413-419, 1972 |
| 1972 | Japan (Sapporo) | M. Oshimura | Chromosomal Banding Patterns in Primary and Transplanted Venereal Tumors of the Dog | J Natl Cancer I 51, 4, 1197-1203, 1973, 1972 |
| 1972 | Nigeria (Lagos) | G. O. Esuruoso | Observations in An experimental Veterinary Clinic in the Ikeja Airport Area of Lagos | Nigerian Vet J 1, 7-15, 1972 |
| 1972 | Kenya | D. Rottcher | Clinical features and pathology of transmissible venereal tumours in dogs in Kenya | Tierarztl Umschau 27, 235-238, 1972 |
| 1973 | France (Toulouse) | P. Cabanie | Étude Ultrastructurale du Sarcome de Sticker du Chien a Différents Stades de son Évolution | Revue Med Vet, 124, 10, 1239-1253, 1973 |
| 1973 | Mexico (Mexico City) | P. Hernández-Jáuregui | Ultrastructural and Histochemical Pattern of Regressing Canine Venereal Lymphoma After Cyclophosphamide Treatment | J Natl Cancer Inst, 51, 1187-1196, 1973 |
| 1973 | US (Tennessee, Knoxville) | T. J. Yang | Canine Transmissible Venereal Sarcoma: Transplantation Studies in Neonatal and Adult Dogs | J Natl Cancer I 51, 1915-1918, 1973 |
| 1973 | Malaysia | D. Cohen | The Biological Behavior of the Transmissible Venereal Tumor in Immunosupressed Dogs | European J Cancer 9, 253-258, 1973 |
| 1973 | Nigeria (Ibadan) | O. O. Oduye | Metastatic transmissible venereal tumor in dogs | J Small Anim Pract 14, 625-637, 1973 |
| 1974 | Kenya (Kabete) | A. Kimeto | Transmissible Venereal Tumour of Dog in Kenya | B Anim Health Prod Afr 22, 327-329, 1974 |
| 1974 | US (Missouri, Columbia) | J. J Broadhurst | Neoplasms of the reproductive system | Published in R.W. Kirk (ed.), Current Veterinary Therapy, W.B. Saunders, Philadelphia, 928-937, 1974 |
| 1974 | Malaysia | R. B. Epstein | Histocompatibility Typing and Course of Canine Venereal Tumors Transplanted into Unmodified Random Dogs | Cancer Res 34, 788-793, 1974 |
| 1974 | Italy (Perugia) | M. Battistacci | Ricerche ultrastrutturali sul Sarcoma di Sticker | Nuova Vet 50, 226-236, 1974 |

| | | | | |
|---|---|---|---|---|
| 1975 | US (Texas) | J.M. Cockrill | Ultrastructural Characteristiscs of Canine Transmissible Venereal Tumor at Various Stages of Growth and Regression | Am J Vet Res, 36, 5, 577-681, 1975 |
| 1975 | Australia (Alice Springs) | K. B. Locke | Transmissible Venereal Tumour in Dogs in Australia | Aust Vet J 51, 449, 1975 |
| 1975 | Sri Lanka | S.G. Wettimuny | Canine neoplasms in Sri Lanka | Ceylon Vet J 23, 1-7, 1975 |
| 1975 | Italy (Sassari) | P. Muzzetto | Il sarcoma di Sticker nota II. (trattamento chirurgico nella cagna) | Clin Vet 98, 124-128, 1975 |
| 1976 | Russia (Moscow) | N. E. Osipov | Diagnosis and treatment of transmissible sarcoma of dogs | Veterinaria (Moscow) 7, 97-98, 1976 |
| 1976 | Italy (Sicily) | C. Murgia | Clonal Origin and Evolution of a Transmissible Cancer | Cell 126, 3, 477-487, 2006 |
| 1976 | Brazil (Botucatu) | A. C. Alexandrino | Tumor venéreo transmissível em caes na regiao de Botucatu | Arq Esc Vet UFMG 28, 1, 101-104, 1976 |
| 1976 | Croatia (Zagreb) | K. Cermak | Prenosivi venericni tumor pasa | Prax Vet 24, 279-287, 1976 |
| 1977 | Iran (Shiraz) | B. Ivoghli | Canine Transmissible Venereal Tumor in Iran | Vet Pathol 14, 289-290, 1977 |
| 1977 | India (Izzat Nagar) | G. C. Mohanty | Growth and Morphological Characteristics of Canine Venereal Tumor Cells *in vitro* | Vet Pathol 14, 420-425, 1977 |
| 1977 | Kenya (Kabete) | C. G. Ndiritu | Extragenitally located transmissible venereal tumor in dogs | Mod Vet Pract 940-946, 1977 |
| 1977 | US (Tennessee, Knoxville or Connecticut, Storrs) | J. R. Kennedy | Canine transmissible Venereal Sarcoma: Electron Microscopic Changes With Time After Transplantation | Br J Cancer 36, 375-385, 1977 |
| 1977 | Nigeria (Ibadan) | L. Idowu | The chromosomes of the transmissible venereal tumour of dogs in Ibadan, Nigeria | Res Vet Sci 22, 271-273, 1977 |
| 1977 | India (Jabalpur) | S. K. Pandey | Canine transmissible venereal sarcoma: clinical trial with autogenous formolized vaccine | Indian Vet J 54, 852-853, 1977 |
| 1977 | Poland (Warsaw) | A. Wasecki | Zostosowonie preporotu Vinblostin w leczeniu guzów Stickero | Med Weter 33, 142-143, 1977 |
| 1977 | France (Maisons-Alfort) | X. Woimant | Neoplasie venerienne du chien et de la chienne (sarcome de Sticker) | Rec Med Vet 153, 331-338, 1977 |
| 1978 | Israel | D. Cohen | The transmissible venereal tumor of the dog - a naturally occurring allograft? A review. | Isr J Med Sci 14, 1, 14-19, 1978 |
| 1978 | Israel (Be'er Sheva) | D. Cohen | Two Cases of the Transmissible Venereal Tumor of the Dog in Israel | Refuah Vet 35, 12-13, 1978 |
| 1978 | Kenya (Kabete) | J. A. Spence | Metastasis of a transmissible venereal tumour to the pituitary | J Small Anim Pract 19,175-184, 1978 |
| 1978 | US (Georgia, Athens) | E. C. Weir | Extragenital Occurrence of Transmissible Venereal Tumor in the Dog: Literature Review and Case Reports | J Am Anim Hosp Assoc 14, 532-536, 1978 |
| 1979 | US (Georgia, Athens) | J. R. Duncan | Cytology of Canine Cutaneous Round Cell Tumors | Vet Pathol 16, 673-679, 1979 |
| 1980 | Italy (Messina, Sicilia, Calabria, Torino) | P. Ajello | Il tumore di Sticker | Annali Facolta Medicina Veterinaria Mess 17, 289-339, 1980 |
| 1980 | Austria (Vienna) | P. Ajello | Il tumore di Sticker | Annali Facolta Medicina Veterinaria Mess 17, 289-339, 1980 |
| 1980 | US (Georgia, Athens) | N. O. Brown | Chemotherapeutic Management of Transmissible Venereal Tumours in 30 dogs | JAVMA 176, 983-986, 1980 |

| | | | | |
|---|---|---|---|---|
| 1980 | South Africa (Onderstepoort) | I. B. J. van Rensburg | Extragenital malignant transmissible venereal tumour in a bitch | J S Afr Vet Assoc 51, 3, 199-201, 1980 |
| 1980 | Nigeria (Nsukka) | J. O. Omamegbe | The surgical treatment of transmissible venereal tumour (TVT) in male dogs - An Assesment of three surgical methods of treatment | Nigerian Vet J 9:-12, 1980b |
| 1980 | France (Toulouse) | G. Madiot | Traitement medico-chirugical du sarcome de Sticker | Thesis presented at the Veterinary School in Toulouse, 1980 |
| 1981 | US (Connecticut, Storrs) | J.P. Chandler | Canine Transmissible Venereal sarcoma: distribution of T and B lymphocytes in blood, draining lymph nodes and tumours at different stages of growth | Br J Cancer 44, 514-520, 1981 |
| 1982 | US (Georgia, Athens) | C. A. Calvert | Vincristine for treatment of transmissible venereal tumor in the dog: clinical reports | JAVMA 181, 2, 163-164, 1982 |
| 1982 | India (Andhra Pradesh) | Ch. Choudary | Certain Canine Neoplasms Encountered in Andhra Pradesh | Indian Vet J 59, 100-102, 1982 |
| 1982 | US (Pennsylvania, Philadelphia) | D. E. Thrall | Orthovoltage radiotherapy of canine transmissible venereal tumors | Vet Radiol 23, 217-219, 1982 |
| 1982 | Nigeria | E. I. Amber | Canine transmissible venereal tumor: Evaluation of surgical excision of primary and metastatic lesions in Zaria - Nigeria. | J Am Anim Hosp Assoc 18, 350-352, 1982 |
| 1983 | US (Florida, Gainesville) | R. M. Bright | Transmissible venereal tumor of the soft palate in a dog | JAVMA 183, 8, 893-895, 1983 |
| 1983 | US (New York) | C. Thacher | Vulvar and vaginal tumors in the dog: A retrospectve study | JAVMA 183, 6, 690-692, 1983 |
| 1983 | US | H. M. Hayes | Canine Transmissible Venereal Tumor: A Model for Kaposi's Sarcoma? | Am J Epidemiol 117, 1, 108-109, 1983 |
| 1983 | Senegal (Dakar) | R. Parent | Presence of the canine transmissible venereal tumor in the nasal cavity of dogs in the area od Dakar (Senegal) | Can Vet J 24, 287-288, 1983 |
| 1984 | US Virgin Islands | D. L. Hill | Canine Transmissible Venereal Sarcoma: Tumor Cell and Infiltrating Leukocyte Ultrastructure at Different Growth Stages | Vet Pathol 21, 39-45, 1984 |
| 1984 | Nigeria (Ibadan) | A. L. Idowu | A retrospective evaluation of four surgical methods of treating canine transmissible venereal tumour | J Small Anim Pract 25, 193-198 |
| 1985 | Papua New Guinea (Boroko) | A. N. Hamir | Primary penile and nasal transmissible venereal tumours in a dog | Aust Vet J, 62, 12, 430-432, 1985 |
| 1985 | Israel (Be'er Sheva) | D. Cohen | The canine transmissible venereal tumor: a unique result of tumor progression | Adv Cancer Res 43, 75-111, 1985 |
| 1985 | Spain | C. Murgia | Clonal Origin and Evolution of a Transmissible Cancer | Cell 126, 3, 477-487, 2006 |
| 1986 | Nigeria (Zaria) | E.I. Amber | Oronasal transmissible venereal tumor in a dog | Mod Vet Pract, 67, 154, 1986 |
| 1986 | Papua New Guinea (Boroko) | A. N. Hamir | Neoplasms of dogs in Papua New Guinea | Aust Vet J 63, 10, 342-343, 1986 |
| 1986 | India (Ranchi) | L. L. Dass | Malignant Transmissible Venereal Tumor | Canine Pract 13, 3, 15-18, 1986 |
| 1987 | US (Connecticut, Storrs and Illinois, Champaign) | N. Katzir | Common origin of transmissible venereal tumors (TVT) in dogs | Oncogene 1, 445-448, 1987 |
| 1987 | Israel (Be'er Sheva) | N. Katzir | Common origin of transmissible venereal tumors (TVT) in dogs | Oncogene 1, 445-449, 1987 |
| 1987 | Nigeria (Nsukka) | B. M. Anene | Common Diseases of Dogs in Nigeria | Zariya Veterinarian 2, 1, 46-55, 1987 |

| | | | | |
|---|---|---|---|---|
| 1987 | Brazil (Jaboticabal) | C.L.M Daleck | Avaliacao de Diferentes Métodos Diagnosticos do Tumor Venéreo Transmissível (T.V.T.) em Caes | ARS Veterinaria 3(2), 187-194, 1987 |
| 1987 | South Africa (Medunsa) | M. I. Vermooten | Canine Transmissible Venereal Tumour (TVT): A Review | J S Afr Vet Assoc 58, 3, 147-150, 1987 |
| 1987 | US (Indiana, West Lafayette) | G. E. Sandusky | Diagnostic Immunohistochemistry of Canine Round Cell Tumors | Vet Pathol 24, 495-499, 1987 |
| 1987 | US Virgin Islands | T. J. Yang | Metastatic transmissible venereal sarcoma in a dog | JAVMA 190, 5, 555-556, 1987 |
| 1987 | US (Connecticut, Storrs) | T. J. Yang | Growth stage dependent expression of MHC antigens on the canine transmissible venereal sarcoma | Br J Cancer 55, 131-134, 1987 |
| 1987 | Brazil (Jaboticabal) | A. A. Camacho | Estudo sobre a eficiencia da vincristine no tratamento de caes com tumor venereo transmissivel | ARS Veterinaria 3(1), 37-42, 1987 |
| 1988 | Greece (Thessaloniki) | C. Boscos | Canine transmissible venereal tumor: clinical observations and treatment | Anim Familiaris 3, 10-15, 1988 |
| 1989 | US (Oklahoma, Stillwater) | R. L. Cowell | Cytology of cutaneous lesions | Vet Clin N Am-Small 19, 4, 769-794, 1989 |
| 1989 | India (Ranchi) | L. L. Dass | Surgical treatment of canine transmissible venereal tumour - a retrospective study | Indian Vet J 66, 255-258, 1989 |
| 1989 | India (Jabalpur) | S. K. Pandey | Incidence, treatment approach and metastasis of canine-transmissible venereal sarcoma | Indian J Anim Sci 59, 5, 510-513, 1989 |
| 1989 | Germany (Munich) | C. Laging | Beobachtungen zum Ubertragbaren Venerischen Tumor (Sticker) beim Hund | Tierarztl Prax 17, 85-87, 1989 |
| 1990 | Nigeria (Zaria) | E. I. Amber | Single-Drug Chemotherapy of Canine Transmissible Venereal Tumor With Cyclophosphamide, Methotrexate, or Vincristine | J Vet Intern Med 4, 144-147, 1990 |
| 1990 | Tanzania Morongo) | E. K. Batamuzi | Canine transmissible tumor in Morongo, Tanzania | Prev Vet J 21, 152-154, 1990 |
| 1990 | US (Alabama, Auburn) | W. W. Miller | Ocular metastasis of a Transmissible Venereal Tumor | Canine Pract 15, 3, 19-21, 1990 |
| 1990 | US | J. E. Moulton | Tumours of Domestic Animals | University of California Press, Berkeley and Los Angeles 10, 498-502, 1990 |
| 1990 | India (Parbhani) | V. S. Panchbhai | Use of autogenous vaccine in transmissible canine venereal tumour | Indian Vet J 67, 983-984, 1990 |
| 1991 | US (Connecticut, Storrs and Illinois, Champaign) | E.N. Amariglio | Identity of rearranged LINE/c-MYC junction sequences specific for the canine transmissble venereal tumor | Proc. Natl. Acad. Sci. USA 88, 8136-8139, 1991 |
| 1991 | Israel (Tel-Aviv, Jerusalem) | E.N. Amariglio | Identity of rearranged LINE/c-MYC junction sequences specific for the canine transmissble venereal tumor | Proc. Natl. Acad. Sci. USA 88, 8136-8140, 1991 |
| 1991 | Tanzania (Morogoro) | E.K. Batamuzi | Anal and perianal transmissible venereal tumour in a bitch | Vet Rec, 129, 556, 1991 |
| 1991 | St. Kitts and Nevis | D. Kroger | An Unusual Presentation of Canine Transmissible Venereal Tumor vol. | Canine Pract 16, 6, 17-21, 1991 |
| 1991 | India (Calcutta) | U. Das | Clinical report on the efficacy of chemotherapy in canine transmissible venereal sarcoma | Indian Vet J 68, 249-252, 1991 |
| 1991 | India (Calcutta) | A. K. Das | A clinical report on the efficacy of vincristine on canine transmissible venereal sarcoma | Indian Vet J 68, 575-576, 1991 |
| 1992 | Tanzania (Morogoro) | E. K. Batamuzi | Risk factors associated with canine transmissible venereal tumour in Tanzania | Prev Vet Med 13, 13-17, 1992 |

| | | | | |
|---|---|---|---|---|
| 1993 | Tanzania (Morogoro) | E.K. Batamuzi | Role of exfoliative cytology in the diagnosis of canine transmissible venereal tumour | J Small Anim Pract 34, 399-401, 1993 |
| 1993 | India (Punjab) | V. K. Gandotra | Occurrence of canine transmissible venereal tumor and evaluation of two treatments | Indian Vet J 70, 854-857, 1993 |
| 1993 | Mexico (Mexico City) | S. E. Morales | The prevalence of transmissible venereal tumor in dogs in Mexico City between 1985-1993 | Vet Mexico 26, 3, 273-275, 1993 |
| 1994 | India (Chennai) | S. Ayyappan | Metastatic transmissible venereal tumour in a dog, a case report | Indian Vet J, 71, 265-266, 1994 |
| 1994 | Spain (Cordoba) | J. Pérez | Primary Extragenital Occurrence of Transmissible Venereal Tumors: Three Case Reports | Canine Pract 19, 1, 7-10, 1994 |
| 1994 | Germany (Giessen) | N. Kirchhof | Spinal metastasis of a canine transmissible tumor | Kleintierpraxis 39, 11, 797-800, 1994 |
| 1994 | India (Mohanpur) | T. B. Sen | Treatment of canine transmissible venereal tumour with cyclophosphamide – a case report | Indian Vet J 71, 1120-1122, 1994 |
| 1995 | Argentina (La Plata) | E. J. Gimeno | Intermediate filament expression and lectin histochemical features of canine transmissible venereal tumour | APMIS 103, 645-650, 1995 |
| 1995 | Spain (Cordoba) | P.J. Ginel | Primary transmissible venereal tumour in the nasal cavity of a dog | Vet Rec 136, 222-223, 1995 |
| 1995 | Italy (Sardinia) | C. Murgia | Clonal Origin and Evolution of a Transmissible Cancer | Cell 126, 3, 477-487, 2006 |
| 1995 | Israel (Jerusalem) | A. Harmelin | Correlation of Ag-NOR protein Measurements with Prognosis in Canine Transmissible Venereal Tumour | J Comp Path 112, 429-433, 1995 |
| 1995 | Zambia (Lusaka) | L. N. Chiti | Anal Transmissible Venereal Tumour in a Dog | Zambian J Vet Sci 1, 1, 21-22, 1995 |
| 1995 | India (Durg) | S. K. Maiti | Therapeutic management of transmissible venereal tumor with vincristine in a dog – a case report | Indian Vet J 72, 614-615, 1995 |
| 1996 | Brazil (Belo Horizonte) | R. M. C. Guedes | Extragenital transmissible venereal tumor in a dog. Case report | Arq Bras Med Vet Zootec 48, 3, 369-374, 1996 |
| 1996 | India (Ludhiana) | J. Singh | Clinico-Pathological Studies on the Effect of Different Anti-Neoplastic Chemotherapy Regimens on Transmissible Venereal Tumours in Dogs | Vet Res Commun 20, 71-81, 1996 |
| 1996 | Spain (Cordoba) | E. Mozos | Immunohistochemical Characterization of Canine Transmissible Venereal Tumor | Vet Pathol 33, 257-263, 1996 |
| 1997 | Brazil | C. Murgia | Clonal Origin and Evolution of a Transmissible Cancer | Cell 126, 3, 477-487, 2006 |
| 1997 | India (Ludhiana) | J. Singh | Effect of geriforte supplementation on blood chemistry in transmissible venereal tumor affected dogs treated with vincristine sulphate | Indian Vet J 74, 420-421, 1997 |
| 1998 | US (Texas, College Station) | K.S. Rogers | Transmissible venereal tumor: A Retrospective Study of 29 Cases | J Am Anim Hosp Assoc 34, 463-470, 1998 |
| 1998 | Greece (Thessaloniki) | C. M. Boscos | Ocular involvement of transmissible venereal tumor in a dog | Vet Opthalmol 1, 167-170, 1998 |
| 1998 | Taiwan(Taichung) | S. C. Chang | A clinical study of primary extragenital transmissible venereal tumors in dogs | J Chin Soc Vet Sci 24, 257-263, 1998 |
| 1998 | Brazil (Jaboticabal) | R. A. Sobral | Occurrence of canine transmissible venereal tumor in dogs from the Jaboticabal region, Brazil | Ars Veterinaria 14, 1, 1-10, 1998 |
| 1998 | Canada (Quebec) - imported from Venezuela | I. Mikaelian | Transmissible venereal tumor: A consequence of sex tourism in a dog | Can Vet J 39, 591, 1998 |

| | | | | |
|---|---|---|---|---|
| 1998 | Spain (Cordoba) | J. Pérez | Immunohistochemical study of the local inflammatory infiltrate in spontaneous canine transmissible vnereal tumour at different stages of growth | Vet Immunol Immunop 64, 133-147, 1998 |
| 1998 | Greece (Thessaloniki) | P. Saratsis | Semen quality during Vincristine treatment in dogs with transmissible venereal tumor | Theriogenology 53, 1185-1192, 1998 |
| 1999 | Greece (Thessaloniki) | C. M. Boscos | Cutaneous Involvement of TVT in Dogs: A Report of Two Cases | Canine Pract 24, 4, 6-11, 1999 |
| 1999 | US (Georgia) | C. Murgia | Clonal Origin and Evolution of a Transmissible Cancer | Cell 126, 3, 477-487, 2006 |
| 2000 | India | U. Das | Review of canine transmissible venereal sarcoma. | Vet Res Commun 24: 545-556. |
| 2000 | Turkey (Ankara) | N. Erunal-Maral | Use of Exfoliative Cytology for Diagnosis of Transmissible Venereal Tumour and Controlling the Recovery Period of the Bitch, | Dtsch Tierarzt Wschr 107, 5, 175 -180, 2000 |
| 2000 | Portugal (Lisboa) | A. J. A. Ferreira | Brain and ocular metastases from a transmissble venereal tumour in a dog | J Small Anim Pract 41, 165-168, 2000 |
| 2000 | Chile (Santiago) | C. M. Gonzales | Canine Transmissible Venereal Tumour: A Morphological and Immunohistochemical Study of 11 Tumours in Growth Phase and during Regression after Chemotherapy | J Comp Path 122, 241-248, 2000 |
| 2000 | Brazil (Rio de Janeiro) | J. S. Pereira | Immunohistochemical chracterization of intraocular metastasis of a canine transmissible venereal tumor | Vet Opthalmol 3, 43-47, 2000 |
| 2001 | Brazil (Jaboticabal) | G. N. Rodrigues | Intraocular transmissible venereal tumor in a dog | Ciencia Rural 31, 1, 141-143, 2001 |
| 2001 | Brazil (Alfenas) | M. S. Varaschin | Clinical manifestations and pathological forms of canine venereal tumors in Alfenas, Minas Gerais | Clin Vet 32, 32-38, 2001 |
| 2001 | Italy (Catania) | C. Murgia | Clonal Origin and Evolution of a Transmissible Cancer | Cell 126, 3, 477-487, 2006 |
| 2001 | Greece (Thessaloniki) | L. G. Papazoglou | Primary Intranasal Transmissible Venereal Tumour in the Dog: A Retrospective study of Six Spontaneous Cases | J Vet Med 48, 391-400, 2001 |
| 2001 | Israel (Rehovot) | A. Harmelin | Use of a murine xenograft model for canine transmissible venereal tumor | AJVR 62, 6, 907-910, 2001 |
| 2001 | Pakistan (Faisalabad) | M. Athar | Clinico-therapeutic studies on canine transmissible venereal tumour | Pakistan Vet J 21, 1, 39-43, 2001 |
| 2002 | Italy (Naples) | F. Albanese | Case report: Primary cutaneous extragenital canine transmissible venereal tumour with *Leishmania*-laden neoplastic cells: a further suggestion of histiocytic origin? | Vet Dermatol 13, 243-246, 2002 |
| 2002 | Brazil (Sao Paulo) | C.V.S. Brandao | Transmissible venereal tumour in dogs: a retrospective study of 127 cases (1998-2000) | Rev Educ Contin 5, 25-31, 2002 |
| 2002 | South Korea (Daejeon) | Y.K. Choi | Sequence Analysis of Canine LINE-1 Elements and p53 Gene in Canine Transmissble Venereal Tumor | J Vet Sci 3(4), 285-292, 2002 |
| 2002 | Turkey (Istanbul) | A. Gurel | Transmissible Venereal Tumors Detected in the Extragenital Organs of Dogs | Israel J Vet Med 57, 2, 1-8, 2002 |
| 2002 | Argentina (La Plata) | C. Gobello | Effects of vincristine treatment on semen quality in a dog with a transmissible venereal tumour | J Small Anim Pract 43, 416-417, 2002 |

| | | | | |
|---|---|---|---|---|
| 2002 | Taiwan (Taipei) | Y.-W. Hsiao | Effect of tumor infiltrating lymphocytes on the expression of MHC molecules in canine transmissible venereal tumor cells | Vet Immunol Immunop 87, 19-27, 2002 |
| 2003 | India (Kolkata) | C. Murgia | Clonal Origin and Evolution of a Transmissible Cancer | Cell 126, 3, 477-487, 2006 |
| 2003 | Kenya (Nairobi) | C. Murgia | Clonal Origin and Evolution of a Transmissible Cancer | Cell 126, 3, 477-487, 2006 |
| 2003 | Brazil (Botucatu) | S. Bassani-Silva | Tumor Venéreo Transmissível - Revisao | Revista Petfood & health & care 2, 77-82, 2003 |
| 2003 | Taiwan (Taipei) | K.-W. Liao | Identification of canine transmissible venereal tumor cells using in situ polymerase chain reaction and the stable sequence of the long interspersed nuclear element | J Vet Diagn Invest 15, 399-406, 2003 |
| 2003 | Mexico (Merida - Yucatan) | A. Ortega-Pacheco | Prevalence of transmissible venereal tumor of stray dogs in Merida, Yucatan, Mexico | Rev Biomed 14, 83-87, 2003 |
| 2003 | Italy (Messina) | G. Catone | Canine Transmissible Venereal Tumour Parasitised by *Leishmania infantum* | Vet Res Commun 27, 549-553, 2003 |
| 2004 | Zimbabwe (Harare) | S. Mukaratirwa | Stromal cells and extracellular matrix components in spontaneous canine transmissible venereal tumour at different stages of growth | Histol Histopathol 19, 1117-1123, 2004 |
| 2004 | Zimbabwe (Harare) | S. Mukaratirwa | Canine transmissible venereal tumour: cytogenetic origin, immunophenotype, and immunobiology | Vet Quart 25, 101-111, 2004 |
| 2004 | Mexico (Merida - Yucatan) | C. A. Rebbeck | Origins and Evolution of a Transmissible Cancer | Evolution 63, 2340-2349, 2009 |
| 2004 | South Africa (Cape Town) | C. A. Rebbeck | Origins and Evolution of a Transmissible Cancer | Evolution 63, 2340-2349, 2009 |
| 2004 | Thailand (Nakhon Pathom) | C. A. Rebbeck | Origins and Evolution of a Transmissible Cancer | Evolution 63, 2340-2349, 2009 |
| 2004 | Kenya (Nairobi) | C. A. Rebbeck | Origins and Evolution of a Transmissible Cancer | Evolution 63, 2340-2349, 2009 |
| 2004 | Greece (Thessaloniki) | C. A. Rebbeck | Origins and Evolution of a Transmissible Cancer | Evolution 63, 2340-2349, 2009 |
| 2004 | Malaysia (Selangor) | C. A. Rebbeck | Origins and Evolution of a Transmissible Cancer | Evolution 63, 2340-2349, 2009 |
| 2004 | Greece (Thessaloniki) | C. M. Boscos | Canine TVT - Clinical findings, Diagnosis and Treatment | Proceedings of the 29th World Congress of World Small Animal Association, 2004 |
| 2004 | Brazil (Botucatu) | A. S. Amaral | Cytological diagnosis of transmissible venereal tumor in the Botucatu region, Brazil (descriptive study: 1994-2003) | RPCV 99, 167-171, 2004 |
| 2004 | Taiwan (Taipei) | Y.-W. Hsiao | Tumor-infiltrating Lymphocyte Secretion of IL-6 Antagonizes Tumor-Derived TGFbeta-1 and Restores the Lymphokine-Activated Killing Ability | J Immunol 172, 1508-1514, 2004 |
| 2004 | Nigeria (Ibadan) | M. A. Tella | Complete Regression of Transmissible Venereal Tumor (TVT) in Nigerian Mongrel Dogs with Vincristine Sulphate Chemotherapy | Afr J Biomed Res 7, 133-138, 2004 |
| 2004 | Peru (Lima) | N. Mendoza | Frequency of the Transmissible Venereal Tumour in Dogs: Caseload in the Pathology Laboratory of the National University of San Marcos (Period 1998-2004) | Rev Inv Vet Peru 21, 1, 42-47, 2010 |
| 2005 | Italy (Messina) | C. Murgia | Clonal Origin and Evolution of a Transmissible Cancer | Cell 126, 3, 477-487, 2006 |
| 2005 | Brazil (Botucatu) | M. I. Mello Martins | Canine Transmissible Venereal Tumor: Etiology, Pathology, Diagnosis and Treatment | In: Concannon PW, England G, Ithaca: NY, 2005 |

| | | | | |
|---|---|---|---|---|
| 2005 | Argentina (La Plata) | M. I. Mello Martins | Canine Transmissible Venereal Tumor: Etiology, Pathology, Diagnosis and Treatment | In: Concannon PW, England G, Ithaca: NY, 2005 |
| 2005 | Turkey (Bursa) | D. Nak | A Clinico-pathological Study on the Effect of Vincristine on Transmissible Venereal Tumour in Dogs | J Vet Med A 52, 366-370, 2005 |
| 2006 | Italy (Naples) | F. Albanese | Case report: Extregenital transmissble venereal tumour associated with circulating neoplastic cells in an immunologically compromised dog | Vet Comp Oncol 4, 1, 57-62, 2006 |
| 2006 | Brazil (Sao Paulo) | C. P. de Brito | Immunohistochemical determination of estrogen receptor-alpha in vaginal and tumor tissues of healthy and TVT-affected bitches and their relation to serum concentrations of estradiol-17beta and progesterone | Theriogenology, 2006, 2006 |
| 2006 | Mexico (Merida - Yucatan) | A. Ortega-Pacheco | Pathological Conditions of the Reproductive Organs of Male Stray Dogs in the Tropics: Prevalence, Risk Factors, Morphological Findings and Testosterone Concentrations | Reprod Dom Anim 41, 429-437, 2006 |
| 2006 | Zimbabwe (Harare) | S. Mukaratirwa | Canine Transmissible Venereal Tumour: Assesment of Mast Cell Numbers as Indicators of the Growth Phase | Vet Res Commun 30, 613-621, 2006 |
| 2006 | South Korea (Seoul) | M.-S. Park | Disseminated transmissible venereal tumor in a dog | J Vet Diagn Invest 18, 130-133, 2006 |
| 2006 | Kenya (Nairobi) | T. O. Abuom | Transmissible Venereal Tumor with Subcutaneous and Bone Metastasis in a Dog | Kenya Vet 30, 1, 11-13, 2006 |
| 2006 | Greece (Thessaloniki) | E. Levy | Nasal and oral masses in a dog | Vet Clin Pathol 35, 115-118, 2006 |
| 2006 | Portugal (Vila Verde) | R. Marcos & M. Santos | Cutaneous transmissible venereal tumor without genital involvement in a prepubertal female dog | Vet Clin Pathol 35,106-109, 2006 |
| 2007 | Brazil (Mossoro - RN) | J.S. Batista | Tumor venereo transmissivel canino com localizacao intra-ocular e metastase no baco | Acta Vet Brasilica 1, 1, 45-48, 2007 |
| 2007 | Brazil (Botucatu) | S. Bassani-Silva | Propolis effect *in vitro* on canine Transmissible Venereal Tumor cells | RPCV 102, 261-265, 2007 |
| 2007 | Mexico (Merida - Yucatan) | A. Ortega-Pacheco | Reproductive patterns and reproductive pathologies of stray bitches in the tropics | Theriogenology 67, 382-390 , 2007 |
| 2007 | Brazil (Botucatu) | A. S. Amaral | Cytomorphological characterization of transmissible canine venereal tumor | RPVC 102, 253-260, 2007 |
| 2007 | Brazil (Garca) | K. C. Florentino | Tumor venéreo transmissível cutaneo canino = Relato de Caso | Revista Cientifica Eletonica de Medicina Veterinaria - ISSN, 1679-7353, 2007 |
| 2007 | Nigeria (Zaria) | A. Z. Hassan | Canine Transmissble Venereal Tumor (CTVT) presented to the ABUVTH, Zaria: A 15 Year Review | Presented at the 44th Nigerian Veterinary Medical Association Annual Congress, 22nd-24th October 2007 |
| 2007 | India (Bareilly) | B. C. Nair | A study on spontaneous canine neoplasms in Bareilly, U.P. | Indian J Vet Pathol 31, 2, 166-168, 2007 |
| 2007 | Taiwan (Taipei) | C.-C. Liu | Transient downregulation of monocyte-derived dendritic-cell dfferentiation, function, and survival during tumoral progression and regression in an in vivo canine model of transmissible venereal tumor | Cancer Immunol Immunother 57, 479-491, 2007 |
| 2008 | Italy (Messina) | G. Marino | Alternative Vincristine Treatment in Dogs With Transmissible Venereal Tumour | Veterinaria 22, 4, 13-18, 2008 |

| | | | | |
|---|---|---|---|---|
| 2008 | India (Tamilnadu, Namakkal) | R. Thangathurai | Cytological diagnosis and its histological correlation in canine transmissible venereal tumour | Vet Archiv 78, 5, 369-376, 2008 |
| 2008 | Turkey (Ankara) | A. Bastan | Uterine and Ovarian Metastasis of Transmissible Venereal Tumor in a Bitch | Turk J vet Anim Sci 32, 1, 65-66, 2008 |
| 2008 | Greece (Thessaloniki) | M. E. Mylonakis | A retrospective study of 61 cases of spontaneous canine epistaxis (1998-2001) | J Small Anim Pract 49, 191-196, 2008 |
| 2008 | Brazil (Belo Horizonte) | F. G. A. Santos | Apoptosis in the transplanted canine transmissible venereal tumor during growth and regression phases | Arq Bras Med Vet Zootec 60, 3, 607-612, 2008 |
| 2008 | Mexico (Toluca) | N. Vázquez-Mota | The T963C mutation of TP53 gene does not participate in the clonal origin of canine TVT | Vet Res Commun 32, 187-191, 2008 |
| 2009 | India (Nagpur) | L. A. Khan | Incidence of Venereal Granuloma and its Medicinal Treatment in stray Dogs of Nagpur City | Vet World 2, 1, 13-14, 2009 |
| 2009 | Nigeria (Makurdi) | I.A. Kisani | A case of transmissible venereal tumor in a castrated dog in Benue state, Nigeria | J Anim Plant Sci 2009 5, 527-530, 2009 |
| 2009 | Mexico (Toluca) | A. Sánchez-Servín | TP53 Polymorphisms allow for genetic sub-grouping of the canine transmissible venereal tumor | J Vet Sci 10, 353-355, 2009 |
| 2009 | Brazil (Ilheus) | R. A. Said | Efficacy and side effects of Vincristine sulphate treatment on canine transmissible venereal tumour | Proceedings of the 34th World Small Animal Veterinary Congress WSAVA, 2009, Sao Paulo |
| 2009 | Zimbabwe (Harare) | S. Mukaratirwa | Combination therapy using intratumoral bacillus Calmette-Guerin (BCG) and vincristine in dogs with transmissible venereal tumours: therapeutic efficacy and histological changes. | J S Afr Vet Assoc 80,2, 92-96, 2009 |
| 2010 | Brazil (Sao Paolo) | K. C. Scarpelli | Predictive factors for the regression of canine transmissible venereal tumor during Vincristine therapy | Vet J 183, 3, 362-363, 2010 |
| 2010 | Brazil (Botucatu) | L. F. J. Gaspar | Spontaneous canine transmissible venereal tumor: cell morphology and influence on P-glycoprotein expression | Turk J Vet Anim Sci 34, 5, 447-454, 2010 |
| 2010 | Mexico (Mexico City) | J. C. Cruz | Canine Transmissible Venereal Tumor in the Metropolitan Area of Mexico City | Rev Cientif 20, 4, 362-366, 2010 |
| 2010 | Grenada | R. M. Kabuusu | Risk factors and characteristics of canine transmissible venereal tumours in Grenada, West Indies. | Vet Comp Oncol 8, 1, 50-55, 2010 |
| 2010 | Bangladesh (Dhaka) | M. Tarafder | Prevalence of clinical diseases of pet dogs and risk perception of zoonotic infection by dog owners in Bangladesh | Bangl J Vet Med 8, 2, 163-174, 2010 |
| 2011 | Brazil (Sao Paolo) | D. Stockmann | Detection of the tumour suppressor gene TP53 and expression of p53, Bcl-2 and p63 proteins in canine transmissible venereal tumour | Vet Comp Oncol 9, 4, 251-259, 2011 |
| 2011 | Mexico | L. G. Bautista-Gomez | Analysis of canine transmissible venereal tumor genotypes using the D-loop region of mitochondrial DNA | Genes Genet Syst 86, 351-355, 2011 |
| 2011 | India (Chennai) | T. Sathiamoorthy | Prevalence of reproductive disorders in the stray dogs of Chennai City | JIVA 9, 2, 62-63, 2011 |
| 2011 | India (Tamil Nadu) | M. Selvaraju | Transmissible Venereal Tumor in a German Shepherd Bitch | Indian Pet J (online) 3, 2, ISSN 2230-7613, 2011 |
| 2011 | Grenada | A. Chikweto | Neoplastic and Nonneoplastic Cutaneous Tumors of Dogs in Grenada, West Indies | ISRN Vet Sci Article ID 416435 |

| | | | | |
|---|---|---|---|---|
| 2012 | Italy (Messina) | G. Marino | Clinicopathological study of canine transmissible venereal tumour in leishmaniotic dogs | J Small Anim Pract 53, 323-327, 2012 |
| 2012 | India (Ludhiana) | K. Gupta | Pathological and immunohistochemical studies on rare cases of primary extra-genital transmissible venereal tumours in the mammary gland | Vet Med 57, 4, 198-206, 2012 |
| 2012 | India (Ludhiana) | E. E. Varughese | Successful Management of Metastatic Transmissible Venereal Tumour to Skin of Mammary Region | Reprod Dom Anim 47 (Suppl. 6), 366–369, 2012 |
| 2012 | Brazil (Aracatuba) | J. T. Trevizan | Disseminated Transmissible Venereal Tumour Associated With Leishmaniasis in a Dog | Reprod Dom Anim 47 (Suppl. 6), 356–358, 2012 |
| 2012 | Egypt (Ismailia) | A. M. Ibrahim | Pathology in practice. Transmissible venereal tumor located on the bulbus glandis and body of the penis. | JAVMA 241, 6, 707-709, 2012 |
| 2012 | Turkey (Bursa) | G. R. Ozalp | Vincristine modulates the expression of Ki67 and apoptosis in naturally occurring canine transmissible venereal tumor (TVT) | Biotech Histochem 87, 5, 325-330, 2012 |
| 2013 | Taiwan (Taichung) | Y.-C. Chen | Expression of MAGE-A restricted to testis and ovary or to various cancers in dogs | Vet Immunol Immunopathol 153, 26-34, 2013 |
| 2013 | Paraguay (Asuncion) | K. Kegler | Vaginal Canine Transmissible Venereal Tumour Associated with Intra-tumoural *Leishmania spp*. Amastigotes in an Asymptomatic Female Dog | J Comp Pathol 149, 156-161, 2013 |
| 2013 | Grenada | A. Chikweto | Genital and Extragenital Canine Transmissible Venereal Tumor in Dogs in Grenada, West Indies | OJVM 3, 111-114, 2013 |
| 2013 | Pakistan (Faisalabad) | A. Tariq | Vincristine sulfate: An Effective Drug against Trans-venereal Tumors | IJMVR 3, 11, 62-64, 2013 |
| 2014 | Grenada | J. Milo | A case of ocular canine transmissible venereal tumor | Can Vet J. 55 (2),106, 2014 |
| 2014 | Switzerland | M. Amrein | Vom ewigen Leben eines Hundes | NZZ am Sonntag, Wissen 16 March 2014, 58, 2014 |
| 2014 | Pakistan (Lahore) | F. Awan | Comparison of Different Therapeutic Protocols in the Management of Canine Transmissible Venereal Tumour: Review of 30 Cases | Global Vet 12, 4, 499-503, 2014 |
| 2014 | Brazil (Seropedica) | M. B. Mascarenhas | Immunohistochemical study of genital and extragenital forms of canine transmissible venereal tumor in Brazil | Pesq Vet Bras 34, 3, 250-254 |
| 2014 | Brazil (Curitiba) | D. M. Da Silva | Treatment of canine transmissible venereal tumor using L-asparaginase, prednisone, and surgery in a clinical chemotherapy-resistant case | Turk J Vet Anim Sci 38, 220-223, 2014 |
| 2014 | Brazil (Porto Alegre) | D. G. Gerardi | Expression of P-glycoprotein, multidrug resistance-associated protein, glutathione-S-transferase pi and p53 in canine transmissible venereal tumor | Pesq Vet Brasil 34, 1, 71-78, 2014 |
| 2014 | Bangladesh (Cittagong) | M. S. Islam | Progressive Type of Canine Transmissible venereal Tumor (CTVT) in a male Stray Dog: a Case Report | Res J Vet Pract 2, 4, 70-72, 2014 |
| 2014 | Iran | J. Javanbakht | Canine transmissible venereal tumor and seminoma: a cytohistopathology and chemotherapy study of tumors in the growth phase and during regression after chemotherapy | Tumor Biol, DOI 10.1007/s13277-014-1723-5, 2014 |
