# Supplementary figures and images for: The changing global distribution and prevalence of canine transmissible venereal tumour
Source: BMC Vet Res. 2014 Sep 3;10:168. doi: 10.1186/s12917-014-0168-9 (PMC4152766; doi:10.1186/s12917-014-0168-9)

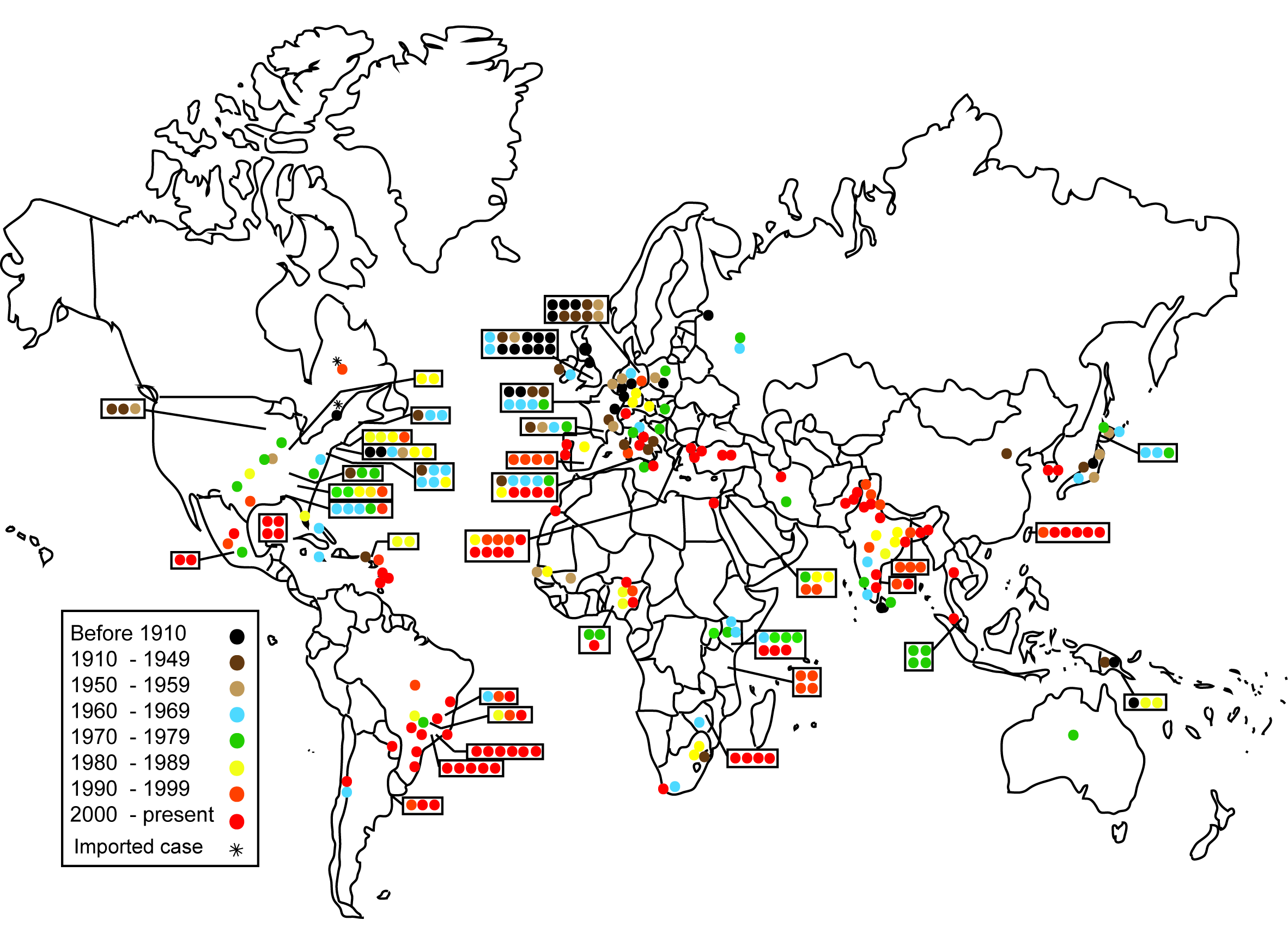

Supplement: Additional file 2 — Global distribution of published reports of CTVT. Locations in which naturally occurring CTVT cases are reported in the published literature are indicated on the map, classified by date of report. Bibliographical information for each case is found in Additional file 1. The two reports in Canada [5],[105] were specified as imported cases from abroad and are marked with *. Map was created using Adobe Illustrator. [file s12917-014-0168-9-S2.tiff]

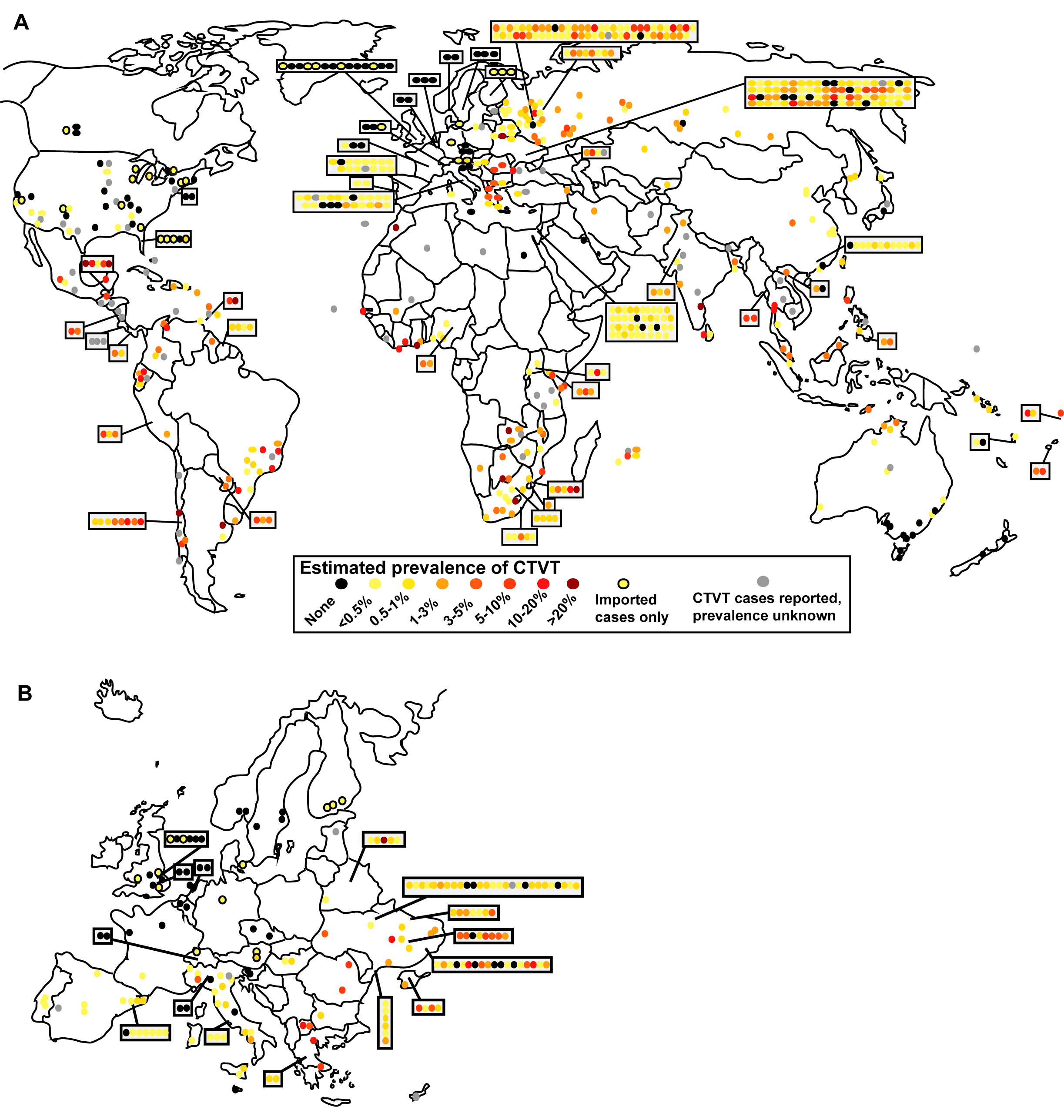

Supplement: Additional file 7 — Worldwide CTVT distribution and prevalence.(A) Map indicating CTVT prevalence estimated by each respondent. Each response is represented by a single coloured dot. Map was created using Adobe Illustrator. (B) Higher magnification map showing detailed distribution of CTVT prevalence estimates in Europe. Map was created using Adobe Illustrator. [file s12917-014-0168-9-S7.tiff]

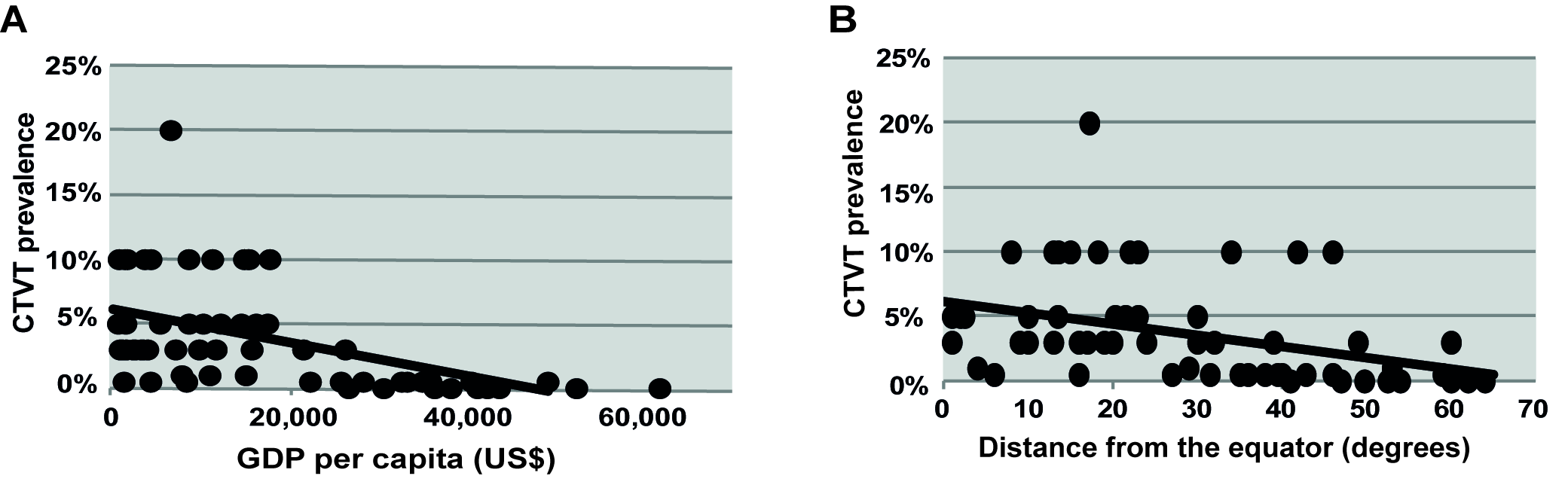

Supplement: Additional file 8 — Socio-economic and climatic factors associated with CTVT prevalence. (A) Relationship between estimated CTVT prevalence and Gross Domestic Product (GDP) per capita values in US dollars. Prevalence values displayed represent the higher limit of each categorical interval. Each dot represents the average of three or more estimated prevalence values received from that country. A line of best fit is displayed (R2 = 0.2543). (B) Relationship between estimated CTVT prevalence and geographical latitude displayed as distance from the equator (in degrees of latitude) measured for the capital city of each country. Prevalence values displayed represent the higher limit of each categorical interval. Each dot represents the average of three or more estimated prevalence values received from that country. A line of best fit is displayed (R2 = 0.1727). [file s12917-014-0168-9-S8.tiff]
