# Supplementary material for: The changing global distribution and prevalence of canine transmissible venereal tumour
Source: BMC Vet Res. 2014 Sep 3;10:168. doi: 10.1186/s12917-014-0168-9 (PMC4152766; doi:10.1186/s12917-014-0168-9)
Supplement: Additional file 3 — Historical reports confirming presence of CTVT in the United Kingdom in the 19th and 20th centuries. The comments were obtained from articles published between 1810 and 1969. [file s12917-014-0168-9-S3.pdf]

Additional file 3. Historical reports confirming presence of CTVT in the United Kingdom in the 19th and 20th centuries.

Year	Town	Author	Comment
1810	UK (London)	D. P. Blaine	two parts only are subject to a cancerous affection [mammary tumour and canine transmissible venereal tumour]
1897	UK (London)	G. B. Smith	Dog A served 12 bitches, eleven of which became infected.
1898	UK (London)	G. B. Smith	Since the beginning of the year 1896 we have had under observation a series of contagious tumours on the genital organs of dogs
1900	UK (London)	F. Hobday	In stud dogs their [CTVT tumours] presence is to be regarded with great suspicion
1902	UK (Leeds)	C. Powell White	I have had the opportunity of examining two similar cases, which present several points of interest. The animals affected were valuable pure-bred bulldogs. The
1905	UK (London)	F. Hobday	Inside the prepuce and on the mucous surface of the penis one meets with another variety, the infective venereal tumour... It appears to be most commonly met with in the bulldog variety, and the author has also seen it in St. Bernards, terriers, poodles and pugs, although there is no reason why it should be confined to these breeds.
1906	UK (London)	F. Hobday	In the vagina of the bitch one frequently meets with a species of ulcerating contagious venereal tumour which is communicable to the male when the animals are used for stud purposes
1907	UK (Edinburgh, London)	H. Wade	The disease known as infective sarcoma is one which occurs in dogs. It is not uncommon… In this country, on the continent of Europe, and in the United States of America it is frequently met with.
1954	UK (London)	E. Cotchin	[tumours occur] less commonly in London dogs
1969	UK (Liverpool)	J. C. Howell	Since 1959 we have diagnosed 13 cases of canine T.V.T.. Eleven of the dogs were owned... Of the other two, one came from Stattford. In only one case was there a history of the dog having lived abroad and this was an animal that had been in Ghana some four to five years before symptoms associated with T.V.T. developed.
1969	UK (London)	R. C. White	I have not seen this condition in this country, but I have examined a number of cases while I was abroad.
