# Supplementary material for: The changing global distribution and prevalence of canine transmissible venereal tumour
Source: BMC Vet Res. 2014 Sep 3;10:168. doi: 10.1186/s12917-014-0168-9 (PMC4152766; doi:10.1186/s12917-014-0168-9)
Supplement: Additional file 4 — Contemporary and historical reports providing a numerical value for CTVT prevalence. [file s12917-014-0168-9-S4.pdf]

Additional file 4. Contemporary and historical reports providing a numerical value for CTVT prevalence.

Year	Location	Prevalence (%)	Details	Author	Title	Reference
1938	France (Toulouse)	**1.8%**	103 CTVT cases in 5643 dogs	R. Lasserre	Recherches sur le Cancer des animaux domestiques	Rev Med Vet 90, 425-451, 1938
1968	Jamaica (Kingston)	**0.8%**	8 CTVT cases in 1000 dogs	M. J. Thorburn	Pathological and cytogenetic observations on the naturally occuring Canine Venereal Tumour in Jamaica (Sticker's tumour)	Brit J Cancer 22, 4, 720-727, 1968
1969	Nigeria (Lagos)	**11.9%**	38 CTVT cases out of 318 dogs presented at the clinic	G. O. Esuruoso	Observations in An experimental Veterinary Clinic in the Ikeja Airport Area of Lagos	Nigerian Vet J 1, 7-15, 1972
1972	Kenya (Kabete)	**1.0%**	Reported prevalence in the population	D. Rottcher	Clinical features and pathology of transmissible venereal tumours in dogs in Kenya	Tierarztl Umschau 27, 235-238, 1972
1974	Kenya (Kabete)	**3.7%**	55 CTVT cases in 1498 post mortem and biopsy cases	A. Kimeto	Transmissible Venereal Tumour of Dog in Kenya	B Anim Health Prod Afr 22, 327-329, 1974
1985	Papua New Guinea (Boroko)	**16.0%**	28 CTVT cases in 170 necropsied dogs (between 1982-83)	A.N. Hamir	Primary penile and nasal transmissible venereal tumours in a dog	Aust Vet J, 62, 12, 430-432, 1985
1986	Papua New Guinea (Boroko)	**19.8%**	53 CTVT cases 268 post mortem examinations	A. N. Hamir	Neoplasms of dogs in Papua New Guinea	Aust Vet J 63, 10, 342-343, 1986
1998	Brazil (Jaboticabal)	**1.9%**	400 cases seen in a 12 year period	R. A. Sobral	Occurrence of canine transmissible venereal tumor in dogs from the Jaboticabal region, Brazil	Ars Veterinaria 14, 1, 1-10, 1998
2003	Mexico (Merida - Yucatan)	**2.6%**	8 CTVT cases in 303 dogs	A. Ortega-Pacheco	Prevalence of transmissible venereal tumor of stray dogs in Merida, Yucatan, Mexico	Rev Biomed 14, 83-87, 2003
2004	Brazil (Botucatu)	**9.9%**	576 CTVT cases out of 5798 cytological exams	A. S. Amaral	Cytological diagnosis of transmissible venereal tumor in the Botucatu region, Brazil (descriptive study: 1994-2003)	RPCV 99, 167-171, 2004

2004	Peru (Lima)	**8.4%**	78 CTVT cases in 925 dogs examined	N. Mendoza	Frequency of the Transmissible Venereal Tumour in Dogs: Caseload in the Pathology Laboratory of the National University of San Marcos (Period 1998-2004)	Rev Inv Vet Peru 21, 1, 42-47, 2010
2006	Mexico (Merida - Yucatan)	**5.4%**	318 post mortem examinations of male stray dogs	A. Ortega-Pacheco	Pathological Conditions of the Reproductive Organs of Male Stray Dogs in the Tropics: Prevalence, Risk Factors, Morphological Findings and Testosterone Concentrations	Reprod Dom Anim 41, 429-437, 2006
2007	Mexico (Merida - Yucatan)	**15.3%**	300 post mortem examinations of female stray dogs	A. Ortega-Pacheco	Reproductive patterns and reproductive pathologies of stray bitches in the tropics	Theriogenology 67, 382-390, 2007
2009	India (Nagpur)	**1.3%**	73 CTVT cases in 5877 dogs examined	L. A. Khan	Incidence of Venereal Granuloma and its Medicinal Treatment in stray Dogs of Nagpur City	Vet World 2, 1, 13-14, 2009
2010	Mexico (Mexico City)	**17.5%**	717 dogs examined	J. C. Cruz	Canine Transmissible Venereal Tumor in the Metropolitan Area of Mexico City	Revista Cientifica 20, 4, 362-366, 2010
2010	Bangladesh (Dhaka)	**0.5%**	20 CTVT cases recorded out of 3670 sick pet dogs presented at the hospital	M. Tarafder	Prevalence of clinical diseases of pet dogs and risk perception of zoonotic infection by dog owners in Bangladesh	Bangl J Vet Med 8, 2, 163-174, 2010
2011	India (Chennai)	**2.7% in males**	18 CTVT cases in 668 dogs examined	T. Sathiamoorthy	Prevalence of reproductive disorders in the stray dogs of Chennai City	JIVA 9, 2, 62-63, 2011
2011	India (Chennai)	**3.9% in females**	24 CTVT cases in 615 dogs examined	T. Sathiamoorthy	Prevalence of reproductive disorders in the stray dogs of Chennai City	JIVA 9, 2, 62-63, 2011
2013	Ecuador (Otavalo)	**2.3%**	5 CTVT cases out of 216 sterilized dogs	A. Strakova and E. P. Murchison	Personal experience	
