# Supplementary material for: The changing global distribution and prevalence of canine transmissible venereal tumour
Source: BMC Vet Res. 2014 Sep 3;10:168. doi: 10.1186/s12917-014-0168-9 (PMC4152766; doi:10.1186/s12917-014-0168-9)
Supplement: Additional file 5 — Text version of CTVT questionnaire used in this study. [file s12917-014-0168-9-S5.pdf]

PROJECT ON CANINE TRANSMISSIBLE VENEREAL TUMOUR WORLDWIDE DISTRIBUTION

Cancer Genome Project, Department of Veterinary Medicine, University of Cambridge

CTVT (also known as Sticker's Sarcoma, Infective Venereal Tumour, TVT, Venereal Sarcoma) is a common disease of dogs and is found worldwide. Primary tumours occur on the external genitalia of both sexes and the disease is usually transmitted during mating.

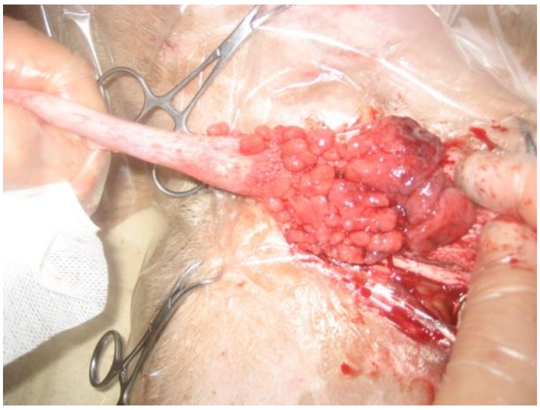

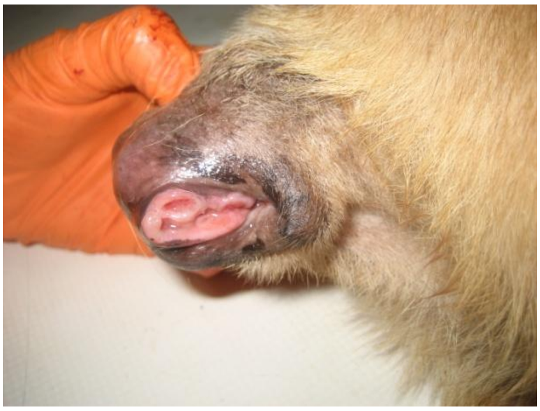

The aim of this research project is to collect data on worldwide prevalence of CTVT, which will help us to identify CTVT risk factors and therefore new possibilities for CTVT prevention. In addition to completing the questionnaire, we would like to invite you to participate in research collaboration by collecting samples and have the opportunity to win a trip to Cambridge. The collection of samples will be used for a genetics study of transmissible cancers and the evolutionary processes which have permitted their emergence and spread worldwide.

All the data will be kept confidential. Your contribution to this project is much appreciated!

YOUR INFORMATION

Name: [ ]

Email address (to keep you updated with project): [ ]

Country: [ ]

Town/village: [ ]

Type of organisation:

- ☐ Private veterinary clinic
- ☐ Veterinary school
- ☐ Pathology laboratory
- ☐ Animal welfare organisation
- ☐ Other, please specify: [ ]

Number of dogs in your area:

- ☐ Fewer dogs than humans
- ☐ One dog per human
- ☐ 2-5 dogs per human
- ☐ > 5 dogs per human

Most dogs in your area are:

- ☐ Spayed/Neutered
- ☐ Entire

If you have extra information, please, comment:

Is there a noticeable population of free roaming dogs in your area?

- ☐ Yes
- ☐ No

If you have extra information, please, comment:

CTVT QUESTIONNAIRE

1) Have you ever seen a case of CTVT?
   - ☐ Yes
   - ☐ No
2) What is your expert opinion on prevalence (% of affected dogs) of CTVT in your region?
   - ☐ None
   - ☐ Less than 0.5%
   - ☐ 0.5-1%
   - ☐ 1 - 3%
   - ☐ 3 - 5%
   - ☐ 5 - 10%
   - ☐ 10 - 20%
   - ☐ More than 20%

If you have more precise data, please comment:

3) How many CTVT case per year do you see?

4) Most of my CTVT cases are from
   - ☐ Urban area
   - ☐ Rural area
   - ☐ Semirural area
   - ☐ Unknown

5) Have you observed CTVT in other regions of your country or the world?

6) Is it noticeably absent from any region in your country?

7) How often does metastasis (lesions in more than one place) occur in the cases you have observed?
   - ☐ 0-5%
   - ☐ 5 -10%
   - ☐ 10 -15%
   - ☐ 15 -20%
   - ☐ More than 20%

What other parts of the body are affected (e.g. skin, lymph nodes,...)?

8) Have you ever observed spontaneous regression of tumours (without treatment)?
   - ☐ Yes
   - ☐ No

If yes, please provide details:

9) Treatment used (tick all that apply)
   - ☐ Vincristine
   - ☐ Doxorubicine
   - ☐ Radiotherapy
   - ☐ Surgery
   - ☐ No treatment
   - ☐ Other

If other, please specify:

10) What proportion of tumours goes into complete remission after using the selected treatment?
   - ☐ 0-20%
   - ☐ 20-40%
   - ☐ 40-60%
   - ☐ 60-80%
   - ☐ 80-100%

11) Gender of the majority of affected dogs
   - ☐ Male
   - ☐ Female
   - ☐ Equally represented

Comment on percentage of predominant sex:

12) Most of dogs with CTVT are:
   - ☐ Otherwise healthy
   - ☐ Diseased/carrying parasites

   If diseased, what is the most frequent disease/parasite in dogs with CTVT?
   - ☐ Injured
   - ☐ Thin, emaciated

What is the most common disease/parasite in your practice?

13) Comment on any unusual cases of CTVT that you have seen:

14) Have you ever come across CTVT in other species (e.g. fox, wolf, coyote, jackal)?

15) Any other comments:

16) Have you got any PHOTOGRAPHS you could send me?(My email address: ctvt@sanger.ac.uk)

17) Is there a national veterinary organisation in your country?

- ☐ Yes
- ☐ No

If yes, what is its name/contact?

18) WOULD YOU LIKE TO TAKE PART IN RESEARCH COLLABORATION? (All materials required for sampling as well as postage are paid for by us!)

- ☐ No
- ☐ Yes

If yes, please be sure to provide your name and email address at the top of the questionnaire!
