# Supplementary material for: The changing global distribution and prevalence of canine transmissible venereal tumour
Source: BMC Vet Res. 2014 Sep 3;10:168. doi: 10.1186/s12917-014-0168-9 (PMC4152766; doi:10.1186/s12917-014-0168-9)
Supplement: Additional file 6 — Summary of CTVT prevalence data by country. [file s12917-014-0168-9-S6.pdf]

Additional file 6. Summary of CTVT prevalence data by country.

Country	Higher confidence average CTVT prevalence (3 or more responses)	Lower confidence average CTVT prevalence (1-2 responses)	Number of responses
Afghanistan	N/A	No data	1
Albania	N/A	N/A	0
Algeria	N/A	1-3%	1
American Samoa	N/A	3-5%	2
Andorra	N/A	N/A	0
Angola	N/A	N/A	0
Antigua and Barbuda	N/A	N/A	0
Argentina	5-10%	N/A	3
Armenia	N/A	1-3%	1
Australia	Less than 0.5%	N/A	18
Austria	N/A	None	2
Azerbaijan	N/A	N/A	0
Bahamas	N/A	0.5-1%	2
Bahrain	N/A	N/A	0
Bangladesh	1-3%	N/A	4
Barbados	N/A	0.5-1%	1
Belarus	0.5-1%	N/A	9
Belgium	N/A	None	2
Belize	10-20%	N/A	6
Benin	N/A	N/A	0
Bhutan	N/A	N/A	0
Bolivia	N/A	N/A	0
Bosnia and Herzegovina	N/A	N/A	0
Botswana	5-10%	N/A	4
Brazil	1-3%	N/A	10
Brunei	N/A	N/A	0
Bulgaria	N/A	0.5-1%	1
Burkina	N/A	N/A	0
Burma (Myanmar)	N/A	N/A	0
Burundi	N/A	N/A	0
Cambodia	N/A	5-10%	1
Cameroon	N/A	N/A	0

Canada	None	N/A	3
Cape Verde	N/A	N/A	0
Central African Republic	N/A	N/A	0
Chad	N/A	N/A	0
Chile	3-5%	N/A	11
China	Less than 0.5%	N/A	20
Colombia	N/A	1-3%	2
Comoros	N/A	N/A	0
Congo	N/A	N/A	0
Congo, Democratic Republic of	N/A	N/A	0
Costa Rica	3-5%	N/A	3
Croatia	N/A	N/A	0
Cuba	3-5%	N/A	3
Cyprus	N/A	No data	1
Czech Republic	None	N/A	4
Denmark	N/A	None	1
Djibouti	N/A	N/A	0
Dominica	N/A	3-5%	1
Dominican Republic	1-3%	N/A	3
East Timor (Timor-Leste)	N/A	3-5%	1
Ecuador	3-5%	N/A	4
Egypt	N/A	1-3%	1
El Salvador	N/A	N/A	0
Equatorial Guinea	N/A	N/A	0
Eritrea	N/A	N/A	0
Estonia	Less than 0.5%	N/A	3
Ethiopia	N/A	N/A	0
Fiji	N/A	3-5%	2
Finland	None	N/A	3
France	Less than 0.5%	N/A	4
Gabon	N/A	N/A	0
Gambia	N/A	10-20%	1
Georgia	N/A	N/A	0
Germany	N/A	None	1
Ghana	5-10%	N/A	3
Greece	1-3%	N/A	4
Grenada	N/A	10-20%	2
Guatemala	N/A	N/A	0

Guinea	N/A	N/A	0
Guinea-Bissau	N/A	N/A	0
Guyana	N/A	3-5%	1
Haiti	N/A	N/A	0
Honduras	N/A	5-10%	1
Hungary	N/A	Less than 0.5%	2
Iceland	N/A	N/A	0
India	1-3%	N/A	7
Indonesia	N/A	N/A	0
Iran	N/A	1-3%	1
Iraq	N/A	N/A	0
Ireland	N/A	N/A	0
Israel	Less than 0.5%	N/A	41
Italy	Less than 0.5%	N/A	23
Ivory Coast	N/A	10-20%	2
Jamaica	N/A	N/A	0
Japan	Less than 0.5%	N/A	3
Jordan	N/A	N/A	0
Kazakhstan	N/A	Less than 0.5%	1
Kenya	3-5%	N/A	7
Kiribati	N/A	N/A	0
Korea, North	N/A	N/A	0
Korea, South	N/A	N/A	0
Kuwait	N/A	None	2
Kyrgyzstan	N/A	0.5-1%	1
Laos	N/A	N/A	0
Latvia	N/A	N/A	0
Lebanon	N/A	N/A	0
Lesotho	N/A	More than 20%	1
Liberia	N/A	1-3%	1
Libya	N/A	1-3%	1
Liechtenstein	N/A	N/A	0
Lithuania	N/A	N/A	0
Luxembourg	N/A	N/A	0
Macedonia	5-10%	N/A	3
Madagascar	N/A	N/A	0
Malawi	3-5%	N/A	3
Malaysia	3-5%	N/A	5
Maldives	N/A	N/A	0
Mali	1-3%	N/A	3

Malta	N/A	None	1
Marshall Islands	N/A	N/A	0
Mauritania	N/A	N/A	0
Mauritius	3-5%	N/A	3
Mexico	5-10%	N/A	5
Micronesia	N/A	3-5%	1
Moldova	N/A	N/A	0
Monaco	N/A	N/A	0
Mongolia	N/A	N/A	0
Montenegro	N/A	N/A	0
Morocco	N/A	More than 20%	1
Mozambique	5-10%	N/A	6
Namibia	N/A	1-3%	1
Nauru	N/A	N/A	0
Nepal	N/A	N/A	0
Netherlands	None	N/A	3
New Zealand	None	N/A	3
Nicaragua	5-10%	N/A	3
Niger	N/A	N/A	0
Nigeria	1-3%	N/A	8
Norway	None	N/A	3
Oman	N/A	N/A	0
Pakistan	1-3%	N/A	3
Palau	N/A	N/A	0
Panama	1-3%	N/A	3
Papua New Guinea	N/A	N/A	0
Paraguay	3-5%	N/A	3
Peru	3-5%	N/A	4
Philippines	1-3%	N/A	4
Poland	N/A	N/A	0
Portugal	Less than 0.5%	N/A	4
Puerto Rico	N/A	0.5-1%	1
Qatar	N/A	N/A	0
Reunion	Less than 0.5%	N/A	4
Romania	5-10%	N/A	3
Russian Federation	1-3%	N/A	102
Rwanda	N/A	N/A	0
Saint Kitts and Nevis	N/A	1-3%	2
Saint Lucia	N/A	N/A	0

Saint Vincent and the Grenadines	N/A	1-3%	1
Samoa	5-10%	N/A	3
San Marino	N/A	N/A	0
Sao Tome and Principe	N/A	N/A	0
Saudi Arabia	N/A	N/A	0
Senegal	N/A	0.5-1%	1
Serbia	N/A	N/A	0
Seychelles	N/A	N/A	0
Sierra Leone	N/A	N/A	0
Singapore	N/A	Less than 0.5%	2
Slovakia	N/A	N/A	0
Slovenia	N/A	None	2
Solomon Islands	N/A	0.5-1%	2
Somalia	N/A	N/A	0
South Africa	0.5-1%	N/A	23
South Sudan	N/A	N/A	0
Spain	Less than 0.5%	N/A	14
Sri Lanka	N/A	3-5%	2
Sudan	N/A	N/A	0
Suriname	0.5-1%	N/A	4
Swaziland	N/A	1-3%	2
Sweden	None	N/A	5
Switzerland	None	N/A	4
Syria	N/A	N/A	0
Taiwan	N/A	0.5-1%	1
Tajikistan	N/A	N/A	0
Tanzania	Less than 0.5%	N/A	3
Thailand	5-10%	N/A	6
Togo	N/A	N/A	0
Tonga	N/A	N/A	0
Trinidad and Tobago	N/A	Less than 0.5%	1
Tunisia	N/A	0.5-1%	1
Turkey	3-5%	N/A	3
Turkmenistan	N/A	N/A	0
Tuvalu	N/A	N/A	0
Uganda	1-3%	N/A	4
Ukraine	1-3%	N/A	91
United Arab Emirates	N/A	N/A	0

United Kingdom	None	N/A	15
United States	Less than 0.5%	N/A	35
Uruguay	N/A	1-3%	1
Uzbekistan	N/A	N/A	0
Vanuatu	Less than 0.5%	N/A	3
Vatican City	N/A	N/A	0
Venezuela, Bolivarian Republic of	N/A	5-10%	2
Viet Nam	1-3%	N/A	3
Yemen	N/A	N/A	0
Zambia	5-10%	N/A	3
Zimbabwe	N/A	0.5-1%	2
